# Mechanistic Insights Into the Reduced Pacemaking Rate of the Rabbit Sinoatrial Node During Postnatal Development: A Simulation Study

**DOI:** 10.3389/fphys.2020.547577

**Published:** 2020-11-20

**Authors:** Azzah M. Alghamdi, Craig P. Testrow, Dominic G. Whittaker, Mark R. Boyett, Jules. C. Hancox, Henggui Zhang

**Affiliations:** ^1^Biological Physics Group, School of Physics and Astronomy, The University of Manchester, Manchester, United Kingdom; ^2^Department of Physics, Faculty of Science, University of Jeddah, Jeddah, Saudi Arabia; ^3^Department of Mathematics, University of Nottingham, Nottingham, United Kingdom; ^4^Department of Biomedical Sciences, Faculty of Health and Medical Sciences, University of Copenhagen, Copenhagen, Denmark; ^5^School of Physiology, Pharmacology and Neuroscience, and Cardiovascular Research Laboratories, School of Medical Sciences, University of Bristol, Bristol, United Kingdom; ^6^Peng Cheng Laboratory, Shenzhen, China; ^7^Key Laboratory of Medical Electrophysiology of Ministry of Education and Medical Electrophysiological Key Laboratory of Sichuan Province, Institute of Cardiovascular Research, Southwest Medical University, Luzhou, China

**Keywords:** postnatal development, sinoatrial node, sodium current, funny current, calcium current, pacemaking action potentials, ion channel mechanisms

## Abstract

Marked age- and development- related differences have been observed in morphology and characteristics of action potentials (AP) of neonatal and adult sinoatrial node (SAN) cells. These may be attributable to a different set of ion channel interactions between the different ages. However, the underlying mechanism(s) have yet to be elucidated. The objective of this study was to determine the mechanisms underlying different spontaneous APs and heart rate between neonatal and adult SAN cells of the rabbit heart by biophysical modeling approaches. A mathematical model of neonatal rabbit SAN cells was developed by modifying the current densities and/or kinetics of ion channels and transporters in an adult cell model based on available experimental data obtained from neonatal SAN cells. The single cell models were then incorporated into a multi-cellular, two-dimensional model of the intact SAN-atrium to investigate the functional impact of altered ion channels during maturation on pacemaking electrical activities and their conduction at the tissue level. Effects of the neurotransmitter acetylcholine on the pacemaking activities in neonatal cells were also investigated and compared to those in the adult. Our results showed: (1) the differences in ion channel properties between neonatal and adult SAN cells are able to account for differences in their APs and the heart rate, providing mechanistic insight into understanding the reduced pacemaking rate of the rabbit sinoatrial node during postnatal development; (2) in the 2D model of the intact SAN-atria, it was shown that cellular changes during postnatal development impaired pacemaking activity through increasing the activation time and reducing the conduction velocity across the SAN; (3) the neonatal SAN model, with its faster beating rates, showed a greater sensitivity to parasympathetic modulation in response to acetylcholine than did the adult model. These results provide novel insights into the understanding of the cellular mechanisms underlying the differences in the cardiac pacemaking activities of the neonatal and adult SAN.

## Introduction

In many species maturation leads to a decrease in the heart rate (HR) (Larson et al., 2013). The HR of mammals including humans, rabbits, rats, and dogs (Dobrzynski et al., 2007; Yanni et al., 2010), decreases with age development; in humans, the heart rate range is between approximately 100–150 beats/min in the neonate (Myrnerts Höök et al., 2018), and 60–100 beats/min in the adult (Your heart rate-British Heart Foundation, 2019).

In normal conditions, the HR is determined by the primary pacemaker of the heart, the sinoatrial node (SAN), which possesses intrinsic pacemaking activity (Boyett et al., 2000; Satoh, 2003; Dobrzynski et al., 2007). Spontaneous APs of sinoatrial nodal pacemaker cells are produced by ion channels and ion transporters, as well as by the intracellular Ca^2+^ dynamics in SAN myocytes (Dobrzynski et al., 2007). It has been shown that significant developmental changes in the expression and function of ion channels and other cellular elements may be responsible for a postnatal alteration in the spontaneous activity of the transmembrane potential in single cells isolated from rabbit SAN (Yang et al., 2006; Dobrzynski et al., 2007). As compared with neonatal SAN cells, spontaneous APs in adult SAN cells show a reduction in spontaneous beating rate, increases in action potential duration (APD) and the intrinsic cycle length (CL), and an increasingly negative maximal diastolic potential (MDP) (Roberts et al., 2012; Larson et al., 2013).

A number of studies have investigated the ionic basis of automaticity in the adult SAN and the age-dependent factors responsible for the changes in the intrinsic heart rate (Jose and Collison, 1970; Toda, 1980; Campbell et al., 1992; Opthof, 2000; Baruscotti and Robinson, 2007). However, there have been far fewer studies on the basis of automaticity of the neonate or immature node cells until recently (Yang et al., 2006; Baruscotti and Robinson, 2007; Yang et al., 2002; Baruscotti et al., 1996; Baruscotti et al., 2001; Accili et al., 1997). The mechanism(s) that underlie the different pacemaking activities between the neonatal and the adult SAN are unclear, though a number of recent studies have stated categorically that the ionic basis of automaticity in neonate mammalian cells is distinct from that in the adult (Baruscotti and Robinson, 2007; Jones et al., 2007; Larson et al., 2013).

Prior studies on the developing SAN in rabbits have revealed that the sodium current, *I*_*Na*_ (Baruscotti et al., 1996, 2001), and pacemaker current, *I*_*f*_ (Accili et al., 1997; Yang et al., 2006), are associated with a developmental maturation-dependent decrease of SAN automaticity, as the two currents have been shown to be greater in the neonate than in the adult. In contrast, the opposite was observed for L-type Ca^2+^ current, *I*_*Ca,L*_ (Jones et al., 2007; Protas et al., 2017), as the contribution from this current to the automaticity increased in the central cells of adult SAN in rabbits compared with that in the neonate. This may reflect species differences (Adachi and Shibata, 2013).

Although experimental information regarding developmental maturation-dependent changes in potassium and other transmembrane currents within the sinoatrial node is lacking, there are experimental data that have considered molecular markers of other relevant parameters and highlighted the key differences between neonates and adults, including an increase in the fibroblast content of the node and a lack of expression of connexins (Jones et al., 2004; Baruscotti and Robinson, 2007), each of which may produce alterations to the electrophysiological properties of the cardiac tissues.

It is unclear whether these differences could account for the different pacemaking activities between the neonatal and adult SAN. Computer-based modeling approaches have proved to be powerful tools to gain a further understanding of the behavior and complexity of electrical, mechanical, structural, and genetic mechanisms of the pacemaker activities in healthy animals (Fabbri et al., 2015), and during the development of arrhythmia (Fabbri et al., 2017). They have also offered a means to predict quantitatively the functional roles of altered molecular dynamics and ionic channels in a systematic way that is otherwise difficult to achieve in an experimental setting at the level of ion channels, cells and tissues (Roberts et al., 2012). These models have usually been constructed and validated against experimental data, and so these approaches are considered reliable (Wilders, 2007).

This study was undertaken to provide a framework in which to analyze the ionic mechanisms underlying the changes in cardiac pacemaking action potentials at the single cell level during the developmental maturation of the rabbit SAN. We have combined different experimental findings from voltage clamp experiments with those on gene expression of the ion-channel currents. Through simulations, we first investigated how changes in the expression and function of different ion channels can contribute to the alteration of the spontaneous AP waveform of the SAN cells and therefore slow spontaneous rate at the single-cell level of the adult heart. This was done by simulating the integral effect of remodeled ion channels on the pacemaking rate and AP characteristics, which were validated by comparing the simulation results to prior experimental data. Then, further mechanistic analysis was conducted to investigate the functional role of individual ion channel changes in producing the pacemaking differences between the neonate and the adult. These analyses elucidated the primary ion channel that is responsible for the pacemaking difference between the neonatal and the adult pacemakers, adding new mechanistic insight into the understanding of neonatal and adult pacemakers to previous experimental studies. In addition, we have updated a 2D model of the intact SAN and surrounding atrial tissue to determine the functional impact of development-dependent changes in electrical coupling through connexins on the initiation and conduction of SAN APs, and their conduction into the atrium.

## Materials and Methods

A framework for investigating the underlying mechanism of fast heart rhythm in the neonate rabbit SA node cells arising from ion channel remodeling was developed by updating: (1) the electrophysiologically detailed central and peripheral SAN cell models developed by Zhang et al. (2000) at the single cell level; (2) a 2D anatomical model of the intact SAN-atrium tissue developed by Butters et al. (2010; Bai et al., 2017), which incorporated tissue geometry for SAN and the right atrium (RA), including the crista terminalis (CT) (Dobrzynski et al., 2005). These models represented the adult rabbit. Therefore, based on the literature review of the ion channel properties of neonate heart as summarized in Table 1, both models were modified at the single cell and tissue level for two age groups: neonate and adult. In simulations, we did not include postnatal development changes in the intracellular Ca^2+^ handling because the experimental data of Allah et al. (2011) showed no significant change of Ca^2+^ uptake and Ca^2+^ release proteins, as determined by RyR and SERCA2a expression levels, between the neonatal and adult SAN.

**TABLE 1:** Summary of differences in current densities and kinetic parameters of ion channels between newborn and adult central SAN cells from multiple species.

Ion channel	Current density (pA/pF)		Midpoint potential for the activation curve (mV)		Midpoint potential for the inactivation curve (mV)		Change in relative abundance of mRNAs for ion channel in central SAN	Species	References
						
	Neonate	Adult	Neonate	Adult	Neonate	Adult			
*I*_*Na*_	68.51 ± 16.0	9.00 ± 0.3	−38 ± 2.2	−37.50 ± 0.9	−89.70 ± 0.7	−93.42 ± 1.9	–	Canine	Protas et al. (2010)
	180.55 ± 16.0	0.00 ± 0.0	−33.9 + 0.7	−23.21 ± 0.8	−63.51 ± 1.0	−61.75 ± 1.1	–	Rabbit	Baruscotti et al. (1996)
	–	–	–		–	–	↑ +64%	Rabbit	Allah et al. (2011)
*I*_*f*_	–	–	−68.5 ± 4.0	−63.20 ± 0.6	–	–	–	Rabbit	Yang et al. (2006)
	0.24 ± 1.5	0.15 ± 0.1	–	–	–	–	–	Rabbit	Accili et al. (1997)
	17.23 ± 6.2	9.60 ± 1.0	−82.9 ± 5.2	−84.13 ± 2.9	–	–	–	Canine	Protas et al. (2010)
	–	–	−104.7 ± 3.1	−110.66 ± 3.2	–		–	Mouse	Larson et al. (2013)
	–		–		–		HCN2↑ +31% HCN4↑ +24%	Rat	Huang et al. (2016)
*I*_*Ca,L*_	17.61 ± 2.5	12.31 ± 1.4	−17.33 ± 1.4	−22.00 ± 0.8	−33.41 ± 1.4	−28.31 ± 1.7	–	Rabbit	Protas et al. (2017)
	–	–	–	–	–	–	↑ +42%	Rabbit	Allah et al. (2011)
	9.02 ± 3.1	4.60 ± 2.6	–	–	–	–	–	Mouse	Larson et al. (2013)
*I*_*Ca,T*_	4.55 ± 0.0	4.52 ± 0.0	−33.00 ± 2.2	−35.11 ± 2.0	−65.00 ± 2.1	−65.09 ± 2.4	–	Rabbit	Protas et al. (2017)
	9.10 ± 2.6	6.36 ± 2.7	–		–	–	–	Mouse	Larson et al. (2013)
*I*_*Kr*_	–		–		–		↑ +36%	Rabbit	Allah et al. (2011)
*I*_*Ks*_	–		–		–		↑ +27%	Rabbit	
*I*_*NaCa*_	–		–		–		↑ +66%	Rabbit	
*I*_*K,ACh*_	–		–		–		↑ +60%	Rabbit	
Cx43	–		–		–		↑ +60%	Guinea-pig	Jones et al. (2007)

*Available data regarding relative abundance of mRNAs that code for some ion channels between neonatal and adult cells are also shown.*

### Model Development at the Single-Cell Level

The single-cell models developed by Zhang et al. (2000), as based on experimental data from isolated rabbit SAN preparations, were used as basal models. In brief, the model described the membrane potential of rabbit SAN cells using Hodgkin-Huxley formulations of ionic currents (see Eq. 1) at body temperature (37°C). The model consisted of 39 coupled ordinary differential equations (ODEs) to describe voltage-gated ion-channel currents, exchanger currents, and an ionic pump (see Eq. 2) for central (cell capacitance of 20 pF) and peripheral (cell capacitance of 65 pF) SAN cells.

(1) $$\frac{dV_{m}}{dt}=-\frac{1}{C_{m}}I_{tot}$$

(2) $$\begin{array}{c} I_{tot}=(I_{Na}+I_{Ca}{,}_{T}+I_{Ca}{,}_{L}+I_{Kr}+I_{Ks}+I_{to}+I_{sus}\\ +I_{f}+I_{b}+I_{NaK}+I_{NaCa}{}_{)} \end{array}$$

In order to model the AP of the SAN cell of neonatal rabbits, the conductances and kinetics of some ionic channels responsible for cellular depolarization and repolarization were adjusted, based on a comprehensive literature review of experiment data on some ion-channel currents, such as, *I*_*Ca*_,_*L*_ and *I*_*f*_, and gene expression levels of ion channels, such as *I*_*Ks*_, *I*_*Kr*_ and *I*_*NaCa*_, on the neonatal rabbit SAN (see Table 1).

The contribution of all or individual remodeled ion channels to the genesis of different pacemaking action potentials between neonatal and adult SAN cells were investigated by integral (inclusive) and exclusive methods. Using the inclusive method, the effect of all experimentally observed changes in different ion channels on the APs was simulated, and results were compared with (validated against) experimental data of the pacemaking rate and the characteristics of APs obtained from experimental studies (Baruscotti et al., 1996; Allah et al., 2011). This method helps to answer whether these changes in ion channels are sufficient to account for the observed differences in pacemaking activity between the neonatal and adult SAN cells. Tables 2, 3 summarize experimental data on the two groups’ current densities and conductances of SAN cells. With the exclusive method, only changes in the selected ion channel were considered in each simulation to investigate the relative role of the remodeled channel in affecting pacemaking activity. Using the exclusive method, the major contributor(s) to the greater heart rate of the neonate SAN cells was determined. In all simulations, the models were solved numerically with a time step of 0.01 ms, which is sufficiently small for stable numerical solutions.

**TABLE 2:** Implemented conductances of different ionic currents in the neonate and adult central SAN cell models based on experimental data as listed in Table 1.

Ion current	Conductance		
	Neonate	Adult	Ratio (neonate/adult)
*g*_*Na*_	0.5350 × 10^–6^ μS	0	∞
*g*_*Ca,L*_	0.841 × 10^–2^ μS	0.58 × 10^–2^ μS	1.45
*g*_*K,r*_	10.839 × 10^–4^ μS	7.97 × 10^–4^ μS	1.34
*g*_*K,s*_	6.57 × 10^–4^ μS	5.18 × 10^–4^ μS	1.27
*g*_*f,Na*_	9.5725 × 10^–4^ μS	5.47 × 10^–4^ μS	1.75
*g*_*f,K*_	9.5725 × 10^–4^ μS	5.47 × 10^–4^ μS	1.75
*K*_*NaCa*_	0.4482 × 10^–5^ nA	0.27 × 10^–5^ nA	1.66

**TABLE 3:** Current densities in the neonate and adult central SAN cell models.

Ion current	Current density, pA/pF		
	Neonate (simulation)	Neonate (experimental)	Adult
*I*_*Na*_	179.56	180.5 ± 16	0.0
*I*_*Ca,L*_	17.58	17.6 ± 2.5	12.23
*I*_*f*_	17.45	17.2 ± 6.2	3.36
*I*_*Ks*_	0.84	–	0.66
*I*_*Kr*_	1.07	–	0.97
*I*_*NaCa*_	0.22	–	0.13

### Modeling *I*_*Na*_ in the Neonatal Rabbit SAN

The sodium-channel current, *I*_*Na*_, is considered to be the main ion channel responsible for the upstroke phase of the APs in non-pacemaking cells (Dobrzynski et al., 2007). However, it is not detected in the central cells of the adult rabbit SAN (Zhang et al., 2000; Dobrzynski et al., 2007). In contrast, according to Baruscotti et al. (1996), voltage clamp measurements from neonatal SAN cells revealed the presence of sodium-channel current. This ion-channel current decreased gradually to disappear 40 days after birth. Of further interest is the difference of *I*_*Na*_ between the neonate and young rabbit SAN cells (Baruscotti et al., 1996). In particular, as compared to the neonate there is ∼76% reduction in the sodium channel current density in the young, which ultimately reduced to zero, together with a rightward shift of the activation curve (midpoint changed by 7 mV in the young) and little or no alteration of the inactivation curve (see Table 1). Consistent with this, quantitative PCR analysis of the mRNA of neonatal and adult SAN rabbit cells showed a relatively greater abundance of the isoforms Nav1.1 and Nav1.5 in the neonatal rabbit SAN cells, which significantly decreased during postnatal development (Allah et al., 2011). It is not yet clear if/how such a decreased *I*_*Na*_ helps to explain the slowing of the heart rate with postnatal development.

**FIGURE 1:**
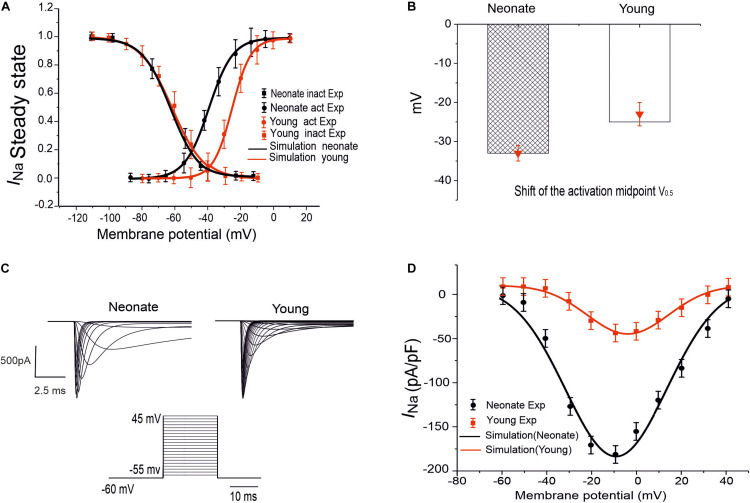
*I*_*Na*_ simulation and validation. **(A)** Steady-state activation and inactivation curves of the *I*_*Na*_ in two groups: neonate (black line) and adult (red line), which are compared with experimental data by Baruscotti et al. (1996). **(B)** Midpoint of the activation curve (V_0.5_) for neonatal and adult. **(C)** Simulated traces of *I*_*Na*_ for neonate and young SAN cells under the voltage-clamp protocol as shown by the inset. **(D)** Simulated I–V relationship and its validation against experimental data by Baruscotti et al. (1996).

To model the postnatal development of the sodium-channel current in rabbit SAN, maturation-dependent *I*_*Na*_ equations were developed based on experimental data from both newborn and young rabbit SAN cells. In the developed model, data on the steady-state activation and inactivation curves for neonate and young rabbit SAN cells reported in Baruscotti et al. (1996) were implemented, taking into account of the shift in the steady-state activation midpoints (as shown in Figures 1A,B). As these experimental data were obtained at room temperature (20–22°C) while the model was developed at body temperature at 37°C, therefore, a Q_1__*O*_ of 1.7 correction of the current density was considered accounting for the temperature difference (Lindblad et al., 1996; Zhang et al., 2000). The maximal channel conductance of the sodium current (*g*_*Na,max*_) was determined by reproducing the experimental data for the I–V relationship (Baruscotti et al., 1996). The developed equations and use of their parameters were validated by the model’s ability to reproduce voltage-clamp data for the *I*_*Na*_ channel, using the same voltage-clamp protocol as used in the experiments. In brief, *I*_*Na*_ was recorded from a holding potential of −65 mV by applying a series of testing potentials that each lasted 10 ms and were varied between −60 mV and +45 mV in 5 mV increments (see Figure 1C). As shown in Table 2, changes in parameters of the age-related *I*_*Na*_ equations were incorporated into the Zhang et al.’s (2000) SAN model. Figure 1D shows the simulated I–V currents for both neonate and young rabbits (black and red lines respectively), which are compared with experimental values (the square points) reported for both neonate and adult rabbits. Simulation data were consistent with the experimental data in showing a peak current density of the neonate I–V curve greater than that of the young, similar to experimental data as shown in Table 2. In sensitivity analysis, a systematic change in the conductance of *I*_*Na*_ was implemented to investigate the role of Ina on the pacemaking CL (see Supplementary Figure S1A for details).

### Modeling *I*_*f*_

The hyperpolarization activated current, *I*_*f*_, is well known as a pacemaking current due to its significant contribution to the pacemaking potential during the phase 4 of the AP of SAN cells (DiFrancesco, 1993; Baruscotti et al., 2005). This ionic channel is permeable to Na^+^ and K^+^ ion fluxes, and comprised of HCN1, HCN2, and HCN4 isoforms, which have been found to be the most abundant isoforms among other subunits (Brioschi et al., 2009). Postnatal changes of this ion channel in rabbit SAN cells were identified experimentally by Accili et al. (1997) and Yang et al. (2006). According to Accili et al. (1997), SAN development affects *I*_*f*_ by decreasing the current density and altering the slope factor of the activation curve of the channel, though without changing the midpoint of the activation curve. The decreased *I*_*f*_ is assumed to result from the change to the cAMP during development. In agreement with this, voltage clamp data recorded by Yang et al. (2006) suggested a smaller *I*_*f*_ in adult SAN cells (12-week-old group) as compared to neonatal SAN cells (2 weeks old) in the rabbit heart. The smaller *I*_*f*_ in the adult group was also associated with a negative shift of the voltage-dependent activation relationship (Table 1). Moreover, quantitative PCR measurements on the mRNA of the HCN subunit confirmed a 69% reduction in HCN4 expression in adult SAN cells compared with the numbers in neonates (Allah et al., 2011). Similar age-induced remodeling of SAN *I*_*f*_ during development was also observed in changes of HCN expression in other species, including rats (Huang et al., 2016), mice (Larson et al., 2013), and canines (Protas et al., 2010). These results indicate that the change in *I*_*f*_ with age may be an important contributor to the declined SAN function and therefore the decreased HR. However, this has not yet been shown explicitly.

**FIGURE 2:**
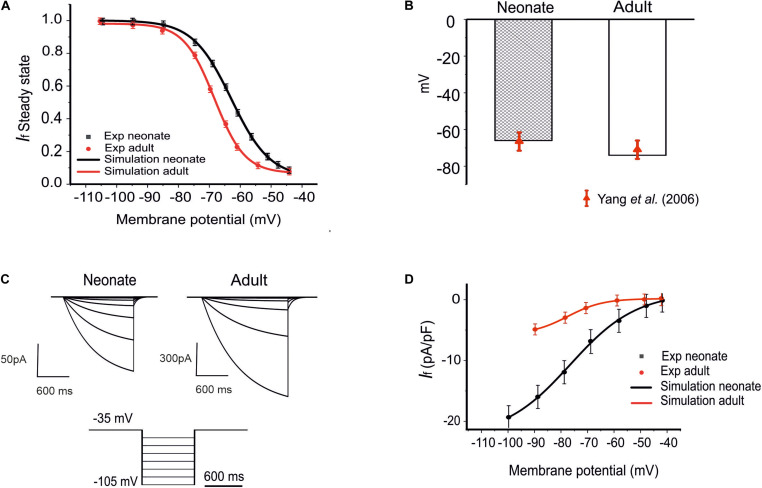
Model of *I*_*f*_ and its comparison to experimental data. **(A)** The steady-state activation curves for the *I*_*f*_ for two age groups: neonates (black line) and adults (red line). **(B)** Shift of the midpoint of the activation relation. **(C)** Simulated time traces of *I*_*f*_ in both neonate and adult SAN cells under the voltage-clamp protocols as shown at the bottom. **(D)** Simulated I–V relationship. Results are validated against experimental data reported by Honjo et al. (1996); Yang et al. (2006), and Protas et al. (2010).

The role of the remodeled *I*_*f*_ on the pacemaking APs of SAN cells was assessed by incorporation of the development maturation-dependent *I*_*f*_ equations into the Zhang et al.’s (2000) model. These maturation-dependent *I*_*f*_ equations were developed based on experimental data from Accili et al. (1997) and Yang et al. (2006), and were validated by their ability to reproduce these experimental data. Results are shown in Figure 2 for neonatal and adult *I*_*f*_, taking into account the maturation-related negative shift of the midpoint of the activation curve by 7 mV as suggested by Yang et al. (2006) (see Figures 2A,B), as well as an increase in the maximal conductances of *g*_*f*_ (e.g., *g*_*f,Na*_, *g*_*f,K*_) by 70%. The time constant for the activation of *I*_*f*_ in neonates was similar to that of adults in the Zhang et al. (2000) model.

The simulated current traces for the *I*_*f*_ current in both neonate and adult SAN cells were obtained under the following voltage-clamp protocols: the *I*_*f*_ current was recorded from a holding potential of −35 mV by applying a series of testing potentials lasting for 600 ms and were varied between −45 mV and −105 mV in 10 mV increments at 36°C, as shown in Figure 2C. The resultant I–V curve (Figure 2D) showed reasonable agreement between the simulation and experimental data obtained for both neonatal and adult cells. The peak current–voltage relationships for *I*_*f*_ from the central SAN cell model in neonates (black line) and adults (red line) are shown in Figure 2D, and these were validated against experimental data reported by Honjo et al. (1996) and Protas et al. (2010). In sensitivity analysis, a systematic change in the conductance for *I*_*f*_ was implemented to investigate the effect of changed *I*_*f*_ on the pacemaking CL (see Supplementary Figure S1B for details).

### Calcium Currents and Remodeling

In the SAN cells, two types of Ca^2+^ current are present: L-Type and T-Type, which are activated at relatively high and low voltages, respectively (Verheijck et al., 1998). The contributions made by the two currents to the pacemaking AP of the rabbit SAN cells have been investigated previously (Dobrzynski et al., 2007). The L-type calcium current was found to be a major contributor to the phase 0 (upstroke) of the APs, either alone in the central cells of adult rabbits’ SAN or in combination with the *I*_*Na*_ current in peripheral SAN cells, in addition to its contribution to the late diastolic depolarization phase of the adult rabbit SAN cells spontaneous AP (Zaza et al., 1996; Verheijck et al., 1999; Mangoni et al., 2003; Baruscotti and Robinson, 2007). A comparative analysis of the postnatal changes of Ca^2+^ ion currents *I*_*Ca,T*_ and *I*_*Ca,L*_ in central cells of the rabbit SAN was carried out by Protas et al. (2017). It was shown that there were no developmental changes in recorded *I*_*Ca,T*_ densities, suggesting that, although it contributes to the automaticity of rabbit SAN cells, *I*_*Ca,T*_ is not a critical determinant of age-dependent changes of the SAN pacemaking.

There is experimental evidence for the reduction of *I*_*Ca,L*_ with postnatal development (Protas et al., 2017); in particular, a 42% reduction in the current density of *I*_*Ca,L*_ has been seen in adults as compared to neonates. However, this was accompanied by a rightward shift of the inactivation curve (midpoint changed by 5 mV in adults), and a leftward shift of the activation curve (midpoint changed by 5 mV in adults), leading to an increase in the window current (the overlap between the activation and inactivation steady-state curves). The increased window current in the adult would suggest a greater contribution of this current to pacemaking in the adult as compared to the neonate. Additional support for this hypothesis comes from the findings of Jones et al. (2007), who observed a progressive loss of Cav1.2 with age in guinea-pig SAN. Moreover, molecular identification of the ion channels at the protein level showed an association between heartbeat decline and decrease in *I*_*Ca,L*_ transcript by 58% in developing SAN rabbit cells (Allah et al., 2011).

**FIGURE 3:**
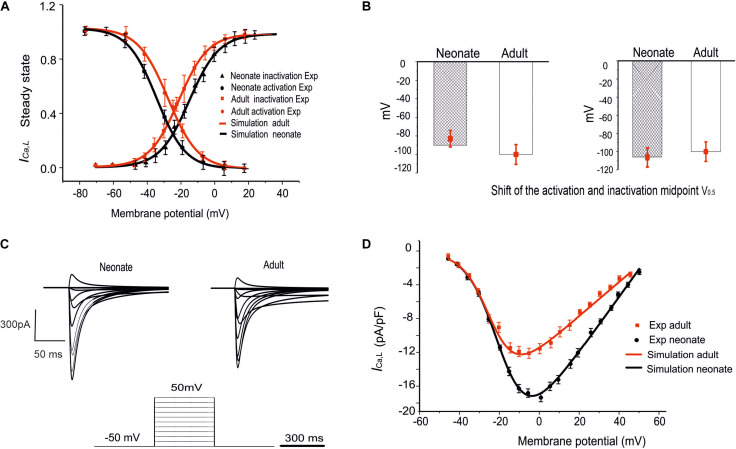
Simulation of *I*_*Ca,L*_ and its validation. **(A)** The fitted steady-state activation and inactivation curves of the *I*_*Ca,L*_ in two age groups: neonates (black line) and adults (red line). **(B)** The bar chart shows the shifted midpoints of activation and inactivation curves. **(C)** The simulated time traces of *I*_*Ca,L*_ in both neonate and adult SAN cells with the voltage-clamp protocols as shown at the bottom of the diagram. **(D)** Simulated I–V relationship. Results validated against experimental data reported by Protas et al. (2017).

To investigate if/how *I*_*Ca,L*_ influences age-dependent changes SAN activity development, a model for neonatal *I*_*Ca,L*_ was developed, based on the above-mentioned experimental data reported by Protas et al. (2017). Figures 3A,B illustrates the simulated steady-state activation and inactivation curves for neonates (black lines) and adults (red lines). The I–V relationship was computed from the model using a voltage clamp protocol with 10 mV incrementing steps between −60 mV and 60 mV of 300 ms duration (see Figure 3C), at a temperature of 36°C. The established I–V relationship curve for the *I*_*Ca,L*_ of neonate and adult rabbit SAN is illustrated in Figure 3D. The magnitude of the neonate (black line) peak current density, 17.30 pA/pF, was considerably larger than the peak current density in adults of 12.32 pA/pF (see Table 3). This result was comparable with values recorded experimentally (Protas et al., 2017). In sensitive analysis, a systematic change in the conductance of *I*_*Ca,L*_ was implemented to study the effect of altered *I*_*CaL*_ in modulating CL (see Supplementary Figure S1C for details).

### *I*_*NaCa*_ and Remodeling

NCX is believed to be functionally important for pacemaking activity through the release of Ca^2+^ from the SR during late diastole and the consequent activation of the inward Na^+^–Ca^2+^ exchange current, *I*_*NaCa*_ (Satoh, 2003). In the SAN, it was observed that the expression of NCX1 for both mRNA and protein levels was more abundant in the neonate than the adult (Allah et al., 2011). In addition, the expression of NCX1 protein was different between the neonate and the adult. This decrease in NCX1 mRNA abundance suggests a possible contribution to the decrease in the heart rate during postnatal development (Allah et al., 2011). Consistent with this, several previous studies have observed a similar postnatal decrease in NCX1 in rabbit ventricles, (Artman, 1992; Artman et al., 1995; Chen et al., 1995; Dan et al., 2007), rabbit whole hearts (Artman, 1992) and human ventricles (Qu et al., 2000). In our model, the *I*_*NaCa*_ of the rabbit SAN cells was considered to be greater in neonates compared to adults; therefore, based on Allah et al. (2011), the magnitude of the *I*_*NaCa*_ current density was adjusted by multiplying the maximal value of the scaling factor for *I*_*NaCa*_ by a ratio of 1.66, as summarized in Tables 1, 2.

### Modeling of the Rapid/Slow Delayed Rectifier K^+^ Currents: *I*_*Kr*_/*I*_*Ks*_

There are two components of the delayed rectifier potassium current in rabbit SAN cells: rapidly activating current (*I*_*Kr*_) and slowly activating current (*I*_*Ks*_). These outward currents are crucial for the repolarization phase of the APs in the SAN. For the neonate condition, limited voltage-clamp experiments for the two currents have been reported in the literature. However, the postnatal developmental changes in the expression of the two ion channels have been characterized by using quantitative PCR, *in situ* hybridization and immunohistochemistry for neonatal (2–7 days of age) and adult (∼6 months of age) New Zealand White rabbits (Allah et al., 2011). The results showed that the mRNA expression of ERG (responsible for *I*_*Kr*_) and both KvLQT1 and minK (responsible for *I*_*Ks*_) were more abundant in the SAN compared to the left ventricle for both neonate and adult cases. It was also found that there was a postnatal reduction in mRNAs associated with delayed rectifier K^+^ channels by 36% for the ERG and 27% for the KvLQT1 in the SAN. Therefore, for the purposes of our simulation, the ratios of the *I*_*Kr*_ and *I*_*Ks*_ maximal conductances of neonate to adult were adjusted by multiplying these ratios by 1.36 and 1.27 as factors in the conductance in the neonatal SAN model, respectively (see Tables 1, 2).

### Acetylcholine-Activated Potassium Current: *I*_*K,ACh*_

Acetylcholine (ACh) is a neurotransmitter which has a chronotropic effect opposite to that of (nor)adrenaline. It is released from the vagal nerve terminals in the SAN, leading to a negative chronotropic effect which appears as a significant reduction in the HR. The effects of ACh on the HR and CL in both age groups for SAN cells in rabbits were simulated by using a formulation of the ACh-activated potassium current, *I*_*K,ACh*_, given by Zhang et al. (2002):

(3) $$\begin{array}{c} I_{K,ACh}=g_{K,ACh^{*}}{\left(\frac{{\left[K\right]}_{e}}{10+{\left[K\right]}_{e}}\right)}^{*}(\left(V_{m}-E_{K}\right)/(1\\ +\text{exp}\left[V_{m}-E_{K}-140\right)F/2.5RT])) \end{array}$$

in which *g*_*K,ACh*_ is the conductance; *I*_*K,ACh*_ is the ionic current; *F* and *R* are the Faraday and universal gas constants, respectively; [K]_*e*_ is the extracellular concentration of K^+^; *V*_*m*_ is the membrane potential; and *E*_*K*_ is the reversal potential for K^+^.

The conductance*g*_*K*,*ACh*_ is given by:

(4) $$g_{K,ACh}=g_{K,AChmax}*jk*\frac{{\left[ACh\right]}^{nK,ACh}}{K_{0.5,K,ACh}^{nK,ACh}+{\left[ACh\right]}^{nK,ACh}}$$

where *j, k* refer to the inactivation variables (fast and slow, respectively); *g*_*K*,*ACh max*_ is the maximum value of *g*_*K*,*ACh*_; *n*_*K*,*ACh*_ is the Hill coefficient; *K_0·5,K,ACh_* is the concentration of ACh that produces a half-maximal activation; and [*ACh*] is the molar concentration of ACh.

The voltage-dependent inactivation variables *j* and *k* are ODEs of the form given in the following equations:

(5) $$\frac{dj}{dt}=\alpha_{j}\left(j-1\right)-\beta_{j}j$$

(6) $$\frac{dk}{dt}=\alpha_{k}\left(1-k\right)-\beta_{k}k$$

In these equations, α_*j*_, β_*j*_, α_*k*_, and β_*k*_ are rate constants. The constants α_*k*_ and α_*j*_ represent voltage-independent constants given by 3.7 s^–1^ and 73.1 s^–1^, respectively. However, the voltage-dependent constants β_*j*_, and β_*k*_ are given by:

(7) $$\beta_{j}=\frac{120}{1+\text{exp}\left(-\left(v_{m}+50\right)/15\right)}$$

(8) $$\beta_{k}=\frac{5.82}{1+\text{exp}\left(-\left(v_{m}+50\right)/15\right)}$$

The equation that describes the change of time dependence of the membrane potential is as follows:

(9) $$\frac{dV_{m}}{dt}=-\frac{I_{tot}+I_{K,ACh}}{C_{m}}$$

in which *I*_*tot*_ is the net magnitude of the various ionic currents.

The incorporation of ACh into our model incorporated two additional effects on ionic currents: a shift in the activation curve for the funny current, *I*_*f*_, and the fractional block of *I*_*Ca,L*_, the L-type Ca^2+^ channel (Zhang et al., 2002).

Acetylcholine causes a shift in the activation curve (mV) of the funny current *I*_*f*_ to a more negative potential as follows:

(10) $$S=S_{max^{*}}\frac{{\left[ACh\right]}^{nf}}{K_{0.5,f}^{nf}\;+\;{\left[ACh\right]}^{nf}}$$

In this equation, *S*_*max*_ refers to the maximum shift of the *I*_*f*_ activation curve; *K*_0.5,f_ is the ACh concentration that produces a half-maximal shift in the funny current activation curve; and *nf* is the Hill coefficient. This was factored into the model by subtracting *s* from the exponent in the Boltzmann equation for the steady-state gating variables of the funny current.

The activated ACh also leads to a partial depression of *I*_*Ca,L*_, which is given by a dimensionless quantity:

(11) $$b=b_{max}^{*}\frac{\left[ACh\right]}{K_{0.5,Ca}+\left[ACh\right]}$$

where *b*_*max*_is the maximum fractional block of *I*_*Ca,L*_, and K_0.5,Ca_ is the concentration of ACh that produces a half-maximal block of *I*_*Ca,L*_. This depression can be included in the model by multiplying the *I*_*Ca,L*_ ionic current by (*1-b*).

For the neonatal condition, it was found in patch clamp experiments that the neonatal SAN cells exhibited particular sensitivity to autonomic stimulation compared with adult SAN cells. Accili et al. (1997) examined the activation curves of the *I*_*f*_ in neonate and adult rabbit cells. It was shown that cells in both groups exhibited similar shifts in the *I*_*f*_ activation in response to maximal concentrations of acetylcholine. Therefore, it was suggested that the greater heart rate and the greater sensitivity of the SAN to ACh was partly the result of the greater magnitude of the hyperpolarization-activated current (*I*_*f*_). Another study (Allah et al., 2011), which measured changes in the mRNA expression of Kir3.1 that is responsible for *I*_*K,ACh*_, found that the relative abundance of mRNAs for Kir3.1 in the SAN was greater (60%) than in the right atrium and left ventricles in the neonates, but not in the adults. In order to model the effect of ACh, the dose dependency of ACh-affected ion channels, including *I*_*K,ACh*_, *I*_*Ca,L*_, and *I*_*f*_, were considered and incorporated into our newly developed neonate (central and peripheral) SAN models, while taking into consideration the fact that *g*_*K,ACh*_ is greater in neonates (60%) by incorporating this percentage as a factor in *g*_*K*,*ACh*_.

### Tissue Model for AP Propagations

In order to investigate the consequences of postnatal changes in different ion-channel properties on the propagation and conduction of excitation waves in the intact SAN-atrium, the 2D anatomical model of the intact SAN-atrium tissue described in previous study (Butters et al., 2010) was implemented. This model considered the heterogeneities of the AP characteristics and the anisotropy of the tissue, including distinct regions of the SAN-atrium, central and peripheral cells of the SAN, which were simulated by Zhang et al. (2000) models. For right atrial cell APs, due to lack of a complete set of experimental data for developing a neonatal model, the model of Aslanidi et al. (2009) for the adult atrial cell was used for both neonatal and adult conditions. The geometry of the 2D model was extracted using histological and immunohistochemistry-imaging data from anatomical models of rabbit SAN cells (Verheijck et al., 1998). The geometry represented the whole intact SAN-atrium meshing from the endo-cardiac surface with a high spatial resolution of 0.04mm to form a Cartesian grid which was divided into 385 × 250 nodes (Dobrzynski et al., 2005; Butters et al., 2010).

In the SAN-atrium tissue model, intercellular electrical coupling *via* connexin between cells is modeled by the diffusive interactions of membrane potentials, which generates the propagation of excitation waves. In the tissue model, such a diffusive interaction of membrane potentials is modeled by a diffusion coefficient (*D*). Since our model represents an intact SAN-atrial tissue is anisotropic, the 2D model took into consideration of regional differences in the intercellular electrical coupling by using a spatially dependent diffusion coefficient, which determines the conduction velocity of excitation propagation within the SAN-atrium tissue. Anisotropic conduction of the excitation waves due to cardiac fiber orientations was modeled by implementing different values of *D* in the longitudinal and transverse directions of the tissue fiber as used in our previous studies (see Supplementary Figure S2A and Eq. 12) (Dobrzynski et al., 2005; Butters et al., 2010).

(12) $$D\left(x\right)=D_{C}+D_{P}.\left(\frac{1}{1+{\text{e}}^{-0.5\left(x-x1\right)}}+\frac{1}{1+{\text{e}}^{0.5\left(x-x2\right)}}\right)$$

where *x* is the horizontal coordinate through the 2D slice, *x*1 and *x*2 approximately correspond to the positions of the SAN boundaries within the tissue, and *D*_*C*_ and *D*_*P*_ are the diffusion coefficients of central and peripheral SAN cells, respectively. The regional differences in cell-membrane capacitance (*C*_*m*_) in the SAN-atrium tissue were based on the study of Zhang et al. (2000) model (Supplementary Figure S2B). The spatial distributions of current densities for different ion channels were correlated with the cell membrane capacitance (*C*_*m*_), which increased gradually from the center to the periphery of the SAN (Zhang et al., 2007).

The 2D model used the monodomain equation to simulate the propagation of the AP and describe the transmembrane potential changes with time. The equation was solved using the finite partial differential equation (PDE) solver with a time step of 0.01 ms and space step of 0.04 mm, which gave accurate numerical solutions (Dobrzynski et al., 2005; Butters et al., 2010).

To investigate the functional impact of postnatal development in the intact SAN-atrium at the tissue level, the 2D model for the SAN-atrium at the tissue level was used to simulate both the neonatal and adult conditions. In the instance of the neonate, the electrophysiology of the cells in the 2D model was adjusted in the same way as for the single-cell level. In addition, changes to intercellular coupling that arose from the connexin remodeling were considered, as explained in the following section. Further, the pacemaking activity of the AP propagation, activation time and conduction velocity were analyzed, as presented in the results section.

### Remodeling of Connexins

Connexins play a critical role in intercellular electrical coupling between the cells through the movement of ion channels and intracellular exchange via the gap junction, as explained above (Boyett et al., 2006). In the SAN tissue, Dobrzynski et al. (2007) found that Cx43 was present in the peripheral cells of adult rabbits. Another study measured the relative abundance of each Cx43, Cx40, and Cx45 in different tissues including the SAN, right atrium and left ventricle of the rabbit heart, from birth to adulthood. These measurements showed that the Cx45 protein was distributed similarly in the different tissues but at decreased levels in the adult; only 45% of that found in adult compared to neonates (Allah et al., 2011). This observation was corroborated by the study of Jones et al. (2004) in guinea-pig SAN for different age groups, which revealed a substantial rearrangement of Cx43 and a 60% decrease in its expression level in adults compared with neonates (see Supplementary Figure S2C). Therefore, in this simulation, the connexin remodeling was achieved by adjustment of the diffusion coefficient value of *D* within the 2D tissue model (see Supplementary Figure S2C).

## Results

### Effects of Ion-Channel Remodeling at the Single Cell Level

**FIGURE 4:**
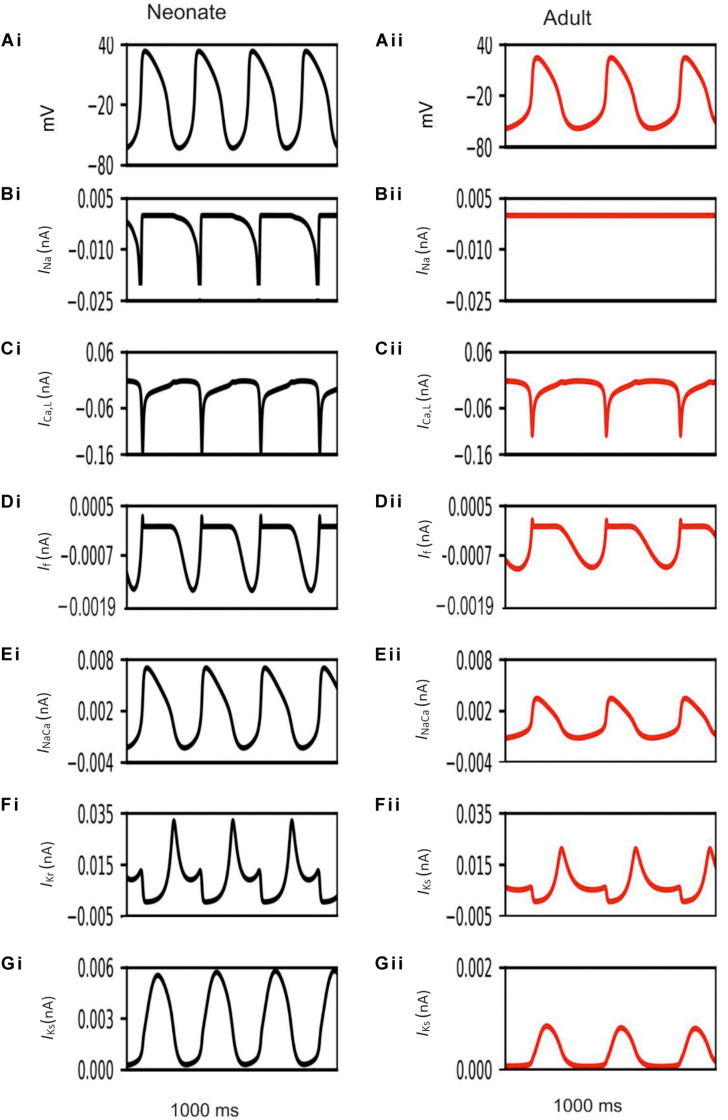
Simulated central SAN action potentials in the neonate (black lines) and adult (red lines) **(Ai,Aii)** and underlying ionic currents *I*_*Na*_, *I*_*Ca,L*_, *I*_*f*_, *I*_*NaCa*_, *I*_*Kr*_ and *I*_*Ks*_ **(Bi–Gii)**.

Development-related remodeling of the different ion channels (*I*_*Na*_, *I*_*Ca,L*_, *I*_*f*_, *I*_*Kr*_, *I*_*Ks*_ and *I*_*NaCa*_) was incorporated into the adult single-cell model of the central SAN developed by Zhang et al. (2000). Figure 4 illustrates the simulated APs and time course of age-remodeled ionic channel currents for the neonatal condition, which were superimposed on those from the adult condition, for the central SAN model. The simulation results showed that the neonatal model reproduces the characteristic AP shapes of neonatal cells as compared with the adult equivalent.

In general, the neonatal APs had faster spontaneous rates, shorter APD and greater amplitudes than those of the adult. The computed CL (the time interval between two successive pacemaking APs) for the neonatal SAN was 277 ms in the neonate and 327 ms in the adult, which showed an 18% prolongation of CL with developmental maturation. The computed maximal upstroke velocity of AP (d*V*/d*t*_*max*_) was 11.9 V/s in the neonate, which was reduced to 2.7 V/s in the adult condition. The greater d*V*/d*t*_*max*_ in the neonatal SAN cells can be attributed to the presence of the *I*_*Na*_. The action potential peak amplitude, PA, of the neonate was greater than that of the adult (32 mV compared with 21 mV) in the central SAN model. These simulation results are consistent with experimental observations, as summarized in Table 4.

**TABLE 4:** AP characteristics of neonatal and adult central SAN cell model.

	References	PA (mV)	CL (ms)	MDP (mV)	d*V*/d*t*_*max*_ (V/s)
Neonate	Experiment (Baruscotti et al., 1996)	40.22 ± 8	280	−59.2 ± 2.2	14 ± 4.5
	Experiment (Allah et al., 2011)	–	246 ± 50	−53.5 ± 2.2	–
	Simulation (our model)	32.22	277	−63	11.99
Adult	Simulation (Zhang et al., 2000)	21.2	327	−57.31	2.76

**FIGURE 5:**
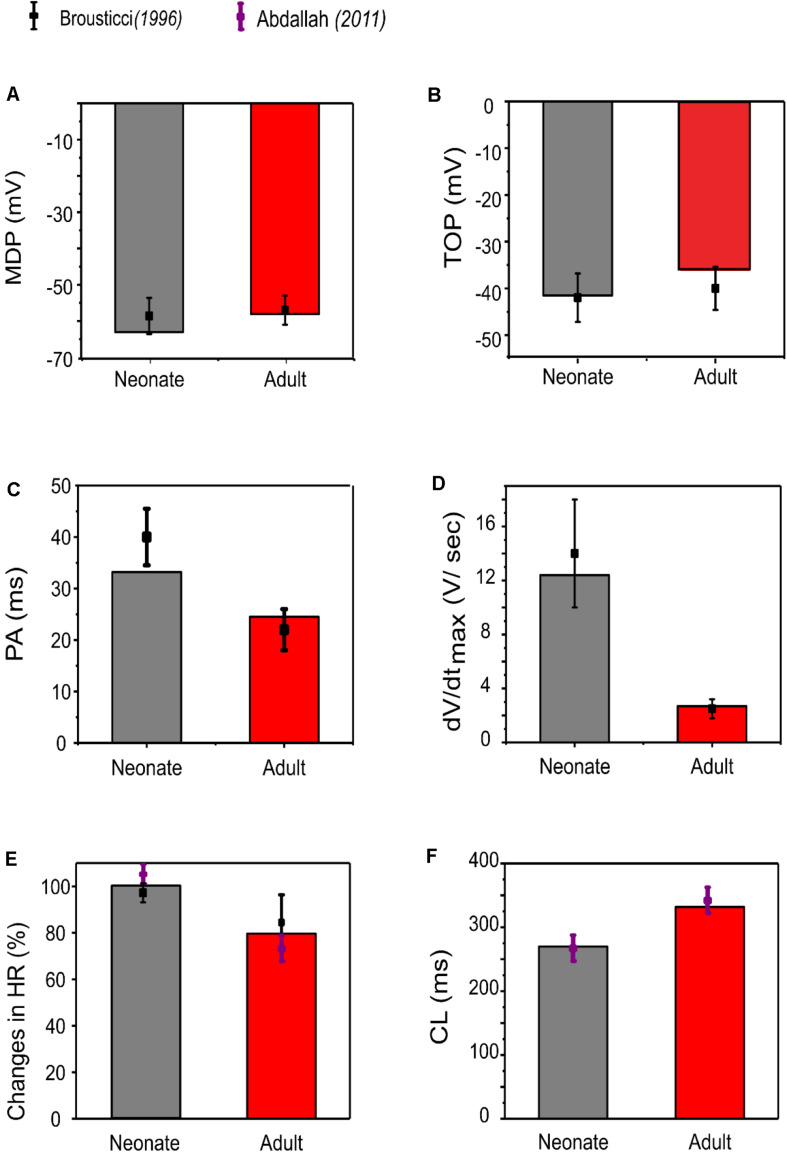
Bar chart comparison of the main AP characteristics of central SAN. **(A)** MDP, **(B)** TOP, **(C)** PA, **(D)** d*V*/d*t*_*max*_, **(E)** HR, and **(F)** CL in neonate (gray) and adult (red) rabbits. All characteristics were compared against experimental values from Baruscotti et al. (1996).

Our simulation showed a reduction of spontaneous AP rate with development, with the measured pacemaking rate of 217 min^–1^ in neonates and 170 min^–1^ in adults (Figure 5). The simulation results were in agreement with the experimental observations, which showed that the heartbeat of the rabbit SAN was around 200 ± 50 min^–1^ for neonates, but then reduced by 21.6% in the adult (Allah et al., 2011). The bar charts in Figure 5 show a comparison of the AP characteristics of the neonatal and adult central SAN from simulations, which quantitatively matched experimental data, validating the model development and showing that the experimentally observed changes in ion channels are sufficient to account for the differences in spontaneous action potentials between the neonatal and the adult SAN cells.

As most available experimental data were acquired from the central SAN cells of neonatal rabbits, data on the peripheral SAN cells were lacking. In simulations, the same modification to ion channels as was implemented in the neonatal central cells was assumed to be applicable to the peripheral model. The resultant APs and the underlying ion channel currents are illustrated in Supplementary Figure S3.

Simulation results showed that the morphology of the neonatal APs of peripheral SAN cells was similar to that of the adult, but with some changed characteristics. The simulated CL in the neonate was smaller than that in the adult: 156 ms compared with 175 ms, indicating 12% reduction in the pacemaking rate by the development. Such heart rate reduction was attributable to the integral actions of altered *I*_*Na*_, *I*_*Ca,L*_, and *I*_*f*_. The measured peak amplitude of the AP in the neonatal condition was greater than that in the adult condition, changing from 31 mV to 23 mV. The reduced AP amplitude by development can be explained by the reduced *I*_*Ca,L*_. In addition, the peripheral cell model considered *I*_*Na*_, but with a greater conductance and changes in the kinetics in neonates as compared with adults. The remodeled *I*_*Na*_ also contributed to the decreased maximal upstroke velocity of APs with developmental maturation. Furthermore, the computed MDP was more negative in the neonatal model than in the adult model, which helped to produce greater *I*_*Na*_, *I*_*Ca,L*_, *I*_*f*_ and *I*_*NaCa*_ during the diastolic phase that help to accelerate the pacemaker depolarization. The development-related remodeling of *I*_*Ks*_, *I*_*Kr*_ and NCX had only small effects on the peripheral AP characteristics, which is discussed in the following section. Characteristics of simulated APs for both peripheral neonatal and adult SAN models are summarized in Supplementary Table S1 and Supplementary Figure S4.

### Functional Role of Individual Remodeling of Ion Channels in the Neonate Condition

**FIGURE 6:**
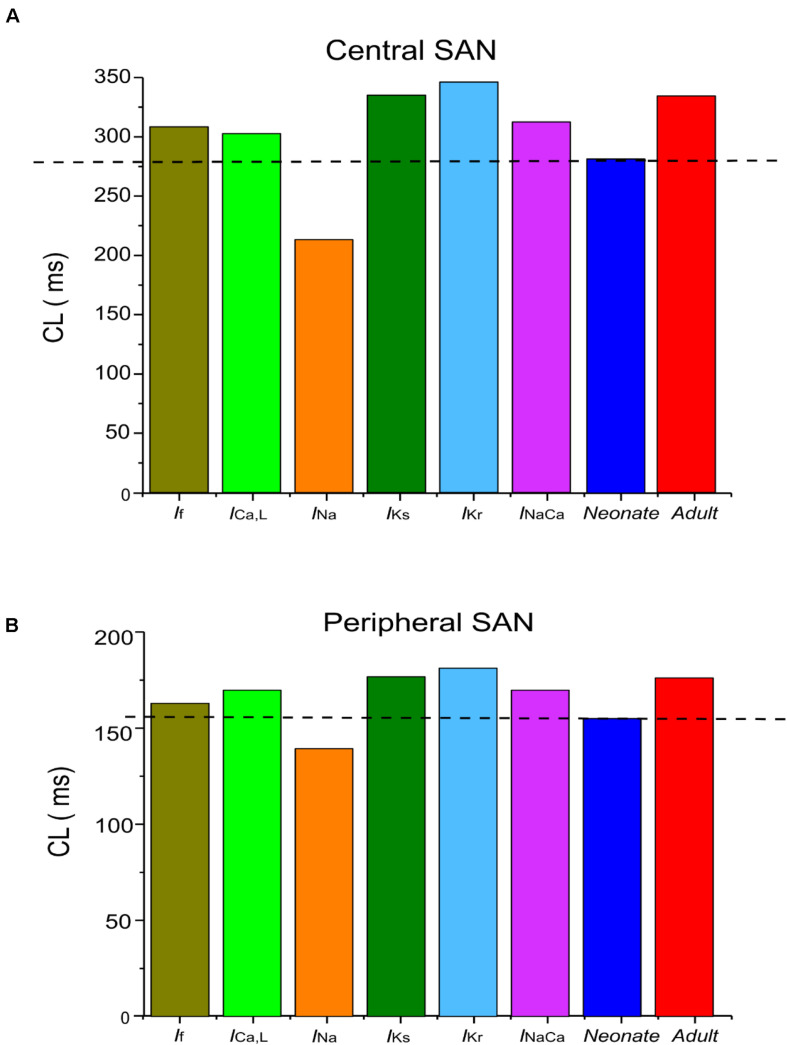
Computed CL from **(A)** central and **(B)** peripheral SAN-cell models, when age-related change to each of the remodeled ionic-channel currents was considered alone.

The central and peripheral cell models were used to investigate the individual roles of each remodeled ion channel in modulating the SAN cell APs, in order to identify the major contributor to the fast neonatal AP rate. This was done by the exclusive method (each individual remodeled ion channel action was considered in turn), with results being compared to those from the inclusive method (i.e., all remodeled ion channel currents were considered in turn) for the neonate case. Results are shown in Figures 6A,B and summarized in Table 5. It was shown that changes of *I*_*f*_, *I*_*CaL*_, *I*_*Na*_ and NCX contributed to a decrease in CL, however, changes in *I*_*Kr*_ and *I*_*Ks*_ contributed to an increase in CL of the neonatal cell model, in both of the central and peripheral cell models.

**TABLE 5:** Effect of individual age-related remodeled ionic-channel currents on the CL of central and peripheral SAN-cell models.

	Center		Periphery	
	CL (ms)	Change in CL compared with adult (ms)	CL (ms)	Change in CL compared with adult (ms)
*I*_*f*_	306	−21	159	−16
*I*_*Ca,L*_	303	−24	170	−5
*I*_*Na*_	204	−123	145	−30
*I*_*Ks*_	327	0	175	0
*I*_*Kr*_	337	+10	177	+2
*I*_*NaCa*_	310	−17	170	−5
Neonate	277	−50	156.23	−18.77
Adult	327	0	175	0

**FIGURE 7:**
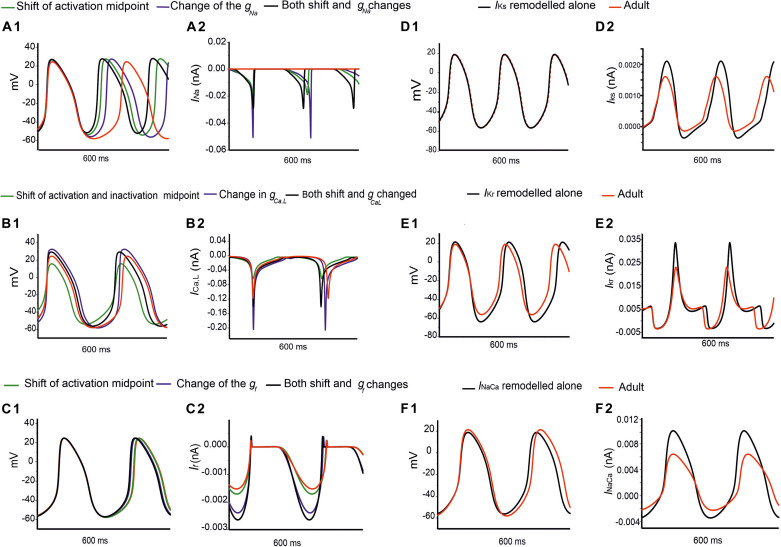
Simulations of the effects of individual remodeled ion channels on modulation of pacemaking APs in the central SAN-cell model. Corresponding action potentials and underlying ionic currents are shown for **(Ai,Aii)** *I*_*Na*_, **(Bi,Bii)** *I*_*Ca,L*_, **(Ci,Cii)** *I*_*f*_, **(Di,Dii)** *I*_*Kr*_, **(Ei,Eii)** *I*_*Ks*_ and **(Fi,Fii)** *I*_*NaCa*_.

Further analysis was conducted to evaluate possible functional roles of the age-dependent differences of *I*_*Na*_ on spontaneous APs, the results are shown in Figures 7Ai,Aii for the central and Supplementary Figures S5Ai,Aii for the peripheral SAN cells. In simulations, when the shift of the activation midpoint was considered alone (the AP and time course of the *I*_*Na*_ represented by green lines compared with the adult red lines), there was a marked reduction in neonatal CL in both central (by 99 ms) and peripheral (by 29 ms) cells compared with CL in the adult. When a reduced *I*_*Na*_ magnitude was considered alone (the AP and time course of *I*_*Na*_ are represented by blue lines compared with the adult red lines), the recorded CL was reduced by 72 ms in the center and 9 ms in the periphery compared with CL values in the adult. When both developmental effects on *I*_*Na*_ were considered (the AP and time course of the *I*_*Na*_ are represented by black lines compared with the adult red lines), the resultant APs were found to be faster than both of the previous changes, with an increase in the diastolic depolarization (phase 0), and a smaller CL by 123 ms in the center and 30 ms in the periphery compared to CL in the adult condition. Thus, a combined effect of the shifted activation curve and reduced current density of *I*_*Na*_ in neonatal SAN cells played a crucial role in influencing AP rate.

Figures 7Bi,Bii and Supplementary Figures S5Bi,Bii show the effect of age-related change of *I*_*Ca,L*_ alone on the pacemaking APs for central and peripheral cell models. In simulations, the shift in the activation and inactivation midpoints alone reduced the CL by 20 ms in the center and 15 ms in the peripheral model as compared with the adult (represented by green lines compared with the adult red lines). When the current density of *I*_*Ca,L*_ was increased (i.e., by modifying the conductance), a decrease in the CL by 12 ms was observed in the center, but an increase of the CL by 20 ms in the peripheral model were observed (represented by blue lines) as compared to the adult APs (represented by red lines), which was due to the regional different contribution of *I*_*Ca,L*_ (Zhang et al., 2000). When the shift in activation/inactivation curves and *I*_*Ca,L*_ densities were considered together, the resultant APs were faster (represented by black lines) (CL being reduced by 24 ms in the center and by 5 ms in the periphery) with a decreased diastolic depolarization phase. This demonstrates that the opposite shift of the steady-state activation and inactivation curves is an important contributor as compared with the current density changes in modulating the pacemaking rate. It was also noted that the PA was greater in the neonate than in the adult by 7 mV in the center and 8 mV in the periphery, indicative of the contribution of the *I*_*Ca,L*_ current to the diastolic depolarization phase (phase 0) and the plateau phase (phase 2).

Figures 7Ci,Cii and Supplementary Figures S5Ci,Cii elucidate the modulatory effect of the *I*_*f*_ current on spontaneous APs in the neonatal SAN. A shift of the steady-state activation curve by −7 mV reduced the CL by 5 ms in the center and 2 ms in the periphery (represented by green lines compared with the adult red lines). An increase in *I*_*f*_ density alone produced a notable decrease in CL: by 16 ms in the center and by 12 ms in the periphery (represented by blue lines). A combined action of the shifted activation curve and increased current density of *I*_*f*_ produced a CL reduction by 21 ms in the center and 16 ms in the peripheral cell models (represented by blue lines).

Figures 7Di,Dii,Ei,Eii,Fi,Fii illustrate the effects of remodeled *I*_*Kr*_, *I*_*Ks*_ and *I*_*NaCa*_ on pacemaking APs of the central cell model, while their effects on the APs of the peripheral cell model are shown in Supplementary Figures S5Di,Dii,Ei,Eii,Fi,Fii. An increase of the *I*_*Ks*_ by 27% as seen in the neonate did not lead to a significant change in the CL or the APs’ morphology. When *I*_*Kr*_ was increased by 36%, there was a CL increase by 10 ms in the center, and by 2 ms in the periphery. An increase of the *I*_*NaCa*_ alone decreased the CL by 17 ms in the central model and caused a small change (5 ms) in the peripheral model. The overall ion channel remodeling effect on the computed CL is summarized in Table 5.

**FIGURE 8:**
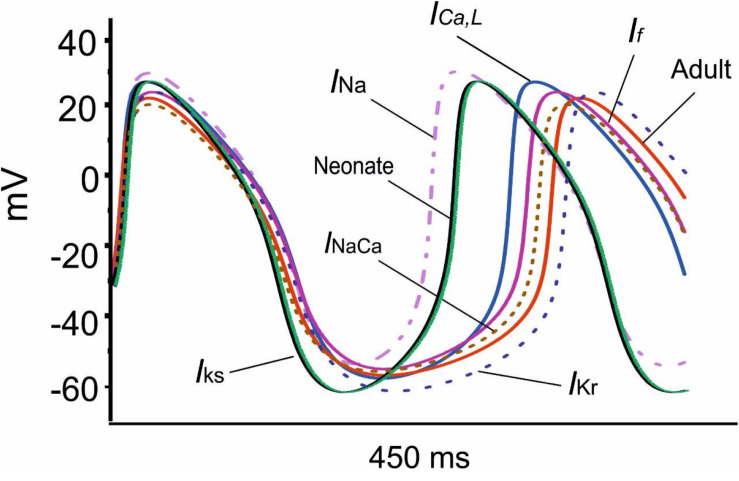
Effect of an individual ion channel remodeling of maturation on modulation of the membrane action potential during the diastolic depolarization phase of SAN cells.

Figure 8 shows overlaid APs in neonatal and adult conditions, together with APs simulated from the adult model with consideration of postnatal development-related changes in *I*_*Na*_, *I*_*CaL*_, *I*_*f*_, *I*_*NaCa*_, *I*_*Kr*_ and *I*_*Ks*_ individually. It was shown that the observed changes in the MDP, AP, ADP between the neonatal and the adult cell models were generated by the integral actions of the considered ion channel and exchanger currents that are responsible for the maturation development of the pacemaking cycle length (CL). It was shown that when the postnatal development change in *I*_*Kr*_ or *I*_*Ks*_ alone was considered, a hyperpolarized maximal diastolic potential was seen, leading to an increase in CL as compared to the adult model, with the effect being more obviously seen in the increased *I*_*Kr*_ case than in the increased *I*_*Ks*_ case.

### Effects of ACh on SAN Cell Activity

**FIGURE 9:**
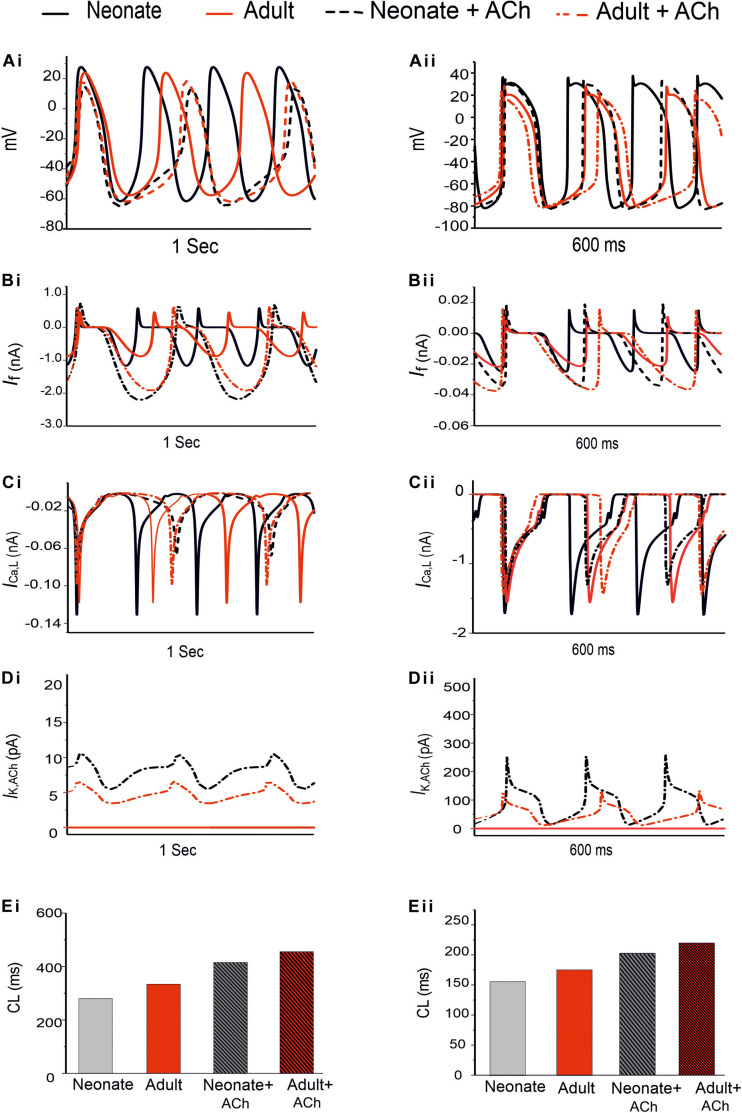
Effect of ACh on the SAN pacemaking APs. Both neonate and adult conditions are shown for the central **(Ai)** and the peripheral SAN cells **(Aii)**; ACh concentration for both conditions was [ACh] = 5 × 10^− 8^ M. **(Bi,Bii)** Time course of *I*_*f*_ current. **(Ci,Cii)** Time course of the *I*_*Ca,L*_ current. **(Di,Dii)** Time course of *I*_*K,Ach*_ current. **(Ei,Eii)** Bar-chart comparison on the computed CL under WT and ACh effects for both cases in the central and peripheral cells.

It is not known whether the negative chronotropic action of ACh is similar between adult and neonatal rabbit SAN cells. Simulations were conducted to address this question, in which actions of ACh on APs were simulated at a physiological concentration of (5 × 10^–8^ M) for the neonate and adult conditions. Results are shown in Figure 9.

In the adult condition, ACh slowed down the pacemaking APs: the measured CL increased from 327 ms to 430 ms (by 30.4%) for the central cells, and from 175 ms to 219 ms (by 23%) for the peripheral cells (Figures 9Ai,Aii). It reduced the PA (from 23.8 mV to 18.38 mV, a decrease of 21.11% in the central cells; from 24.4 mV to 21.5 mV, a decrease of 10.6% in the peripheral cells). It also reduced the APD_50_ (by 13.33% in the center and by 11.9% in the periphery). It hyperpolarized the MDP, which changed from −57 mV to −61 mV in the center and from −80.88 mV to −81.4 mV in the periphery. This resultant negative chronotropic effect of ACh on the APs was attributed to the combination of the activation of *I*_*K,ACh*_, partial depression of *I*_*Ca,L*_ and the *I*_*f*_ activation shift.

Acetylcholine had a greater effect on modulating simulated APs in the neonatal than in the adult SAN cell models, which is consistent with previous experimental observations (Atkins and Marvin, 1989; Zhang et al., 2002). The changes in the CL under the influence of ACh were markedly greater in the neonatal condition: see the bar-chart comparison on the computed CL under WT and ACh effects for both cases in the central and peripheral cells in Figures 9Ei,Eii. This demonstrates that the negative chronotropic effects of ACh were grater in both the center and the peripheral cells of the neonatal models than in the adult ones. The PA changed from 32.5 mV to 14.5 mV (by 55.38%) in the central cells and from 30 mV to 28 mV (by around 13%) in the peripheral cells. There was a reduction in the APD_50_ values by 23.4% in the center and 13% in the periphery. The simulated MDP values for the APs were hyperpolarized from −62.8 mV to −64.6 mV in the center and from −82.04 mV to −83.53 mV in the periphery in the neonate. These changes were 2.8% and 1.8% for the central and peripheral cell models in the neonate condition. All these values are summarized in Table 6.

**TABLE 6:** The negative chronotropic effect of ACh is shown on the pacemaking APs in central and peripheral rabbit SAN cells, at a physiological concentration of 5 × 10^–8^ M for the neonate and adult conditions.

		Neonate			Adult		
		No ACh	ACh 5 × 10^–^^8^ M	Change %	No ACh	ACh 5 × 10^–^^8^ M	Change %
Center	MDP (mV)	−62.08	−64.6	↑2.8	−57	−61	↓7
	PA (mV)	32.5	14.5	↓55.38	23.8	18.38	↓21.11
	CL (ms)	277	403	↑47	327	430	↑30.0
Periphery	MDP (mV)	−82.04	−83.53	↓1.8	−80.88	−81.0	↓2.4
	PA (mV)	30	28	↓13	24.4	21.5	↓10.6
	CL (ms)	156	202.37	↑27	175	219	↑ 23.0

**FIGURE 10:**
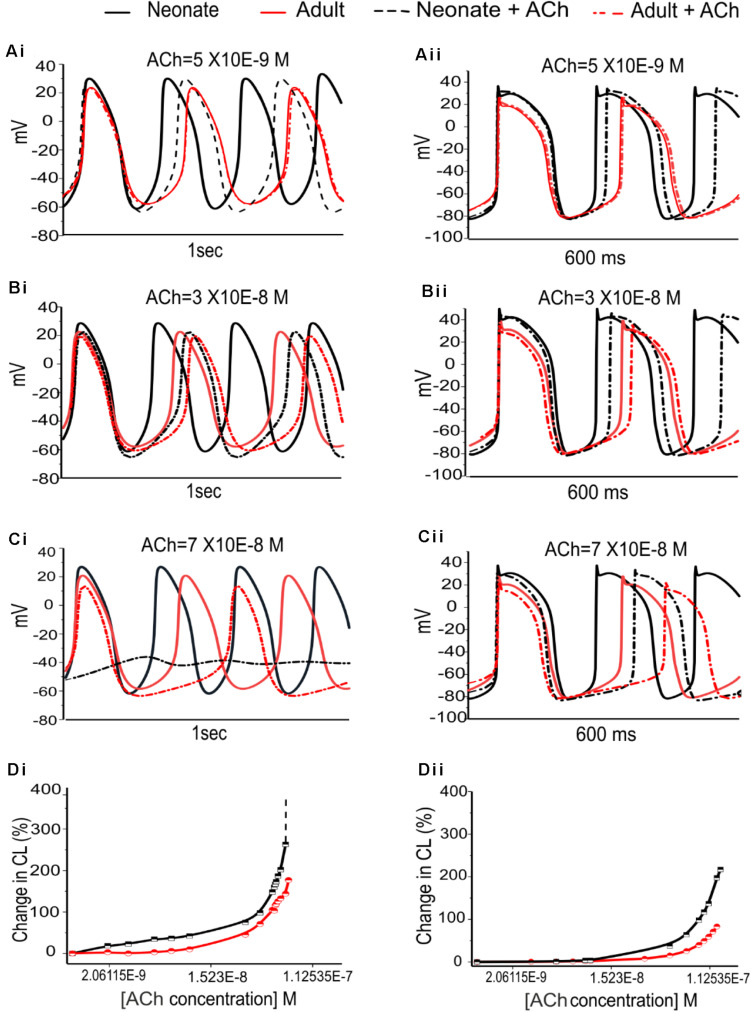
Dose-dependent effects of ACh on spontaneous APs of the SANs for the neonate and adult. **(Ai,Aii)**: At [ACh] = 5 × 10^− 9^ M. **(Bi,Bii)**: At [ACh] = 3 × 10^− 8^ M. **(Ci,Cii)**: At [ACh] = 7 × 10^− 8^ M. **(Di,Dii)**: Dose-dependent effects of ACh on pacemaking CL for the central and peripheral SAN cells, respectively. The black line represents the neonate condition and the red line represents the adult condition.

The dose-dependent effects of ACh on pacemaking APs in developing SAN were also investigated. Figure 10 illustrates the results obtained from the central and peripheral cell models for both age groups under three different “physiological” ACh concentrations. Increase of the ACh concentration resulted in a monotonic increase of CL of the APs for both cell types, with greater effects in the neonate condition. At an ACh concentration of 7.0 × 10^–8^ M (Figures 10Ci,Cii), pacemaking was still occurred in the adult central SAN cells (though with a prolonged CL compared with the control condition) but became quiescent in the neonatal condition. The results for the peripheral cells showed less sensitivity to ACh at a concentration of 7.0 × 10^–8^ M compared with the central model, although the neonate was more affected than the adult. Figures 10Di,Dii summarizes the simulated dose-dependent effect of ACh on the CL recorded in both conditions. The concentration-response relation of the CL shifted leftward with developmental maturation, indicating an amplified effect of ACh on SAN cells in neonates.

**FIGURE 11:**
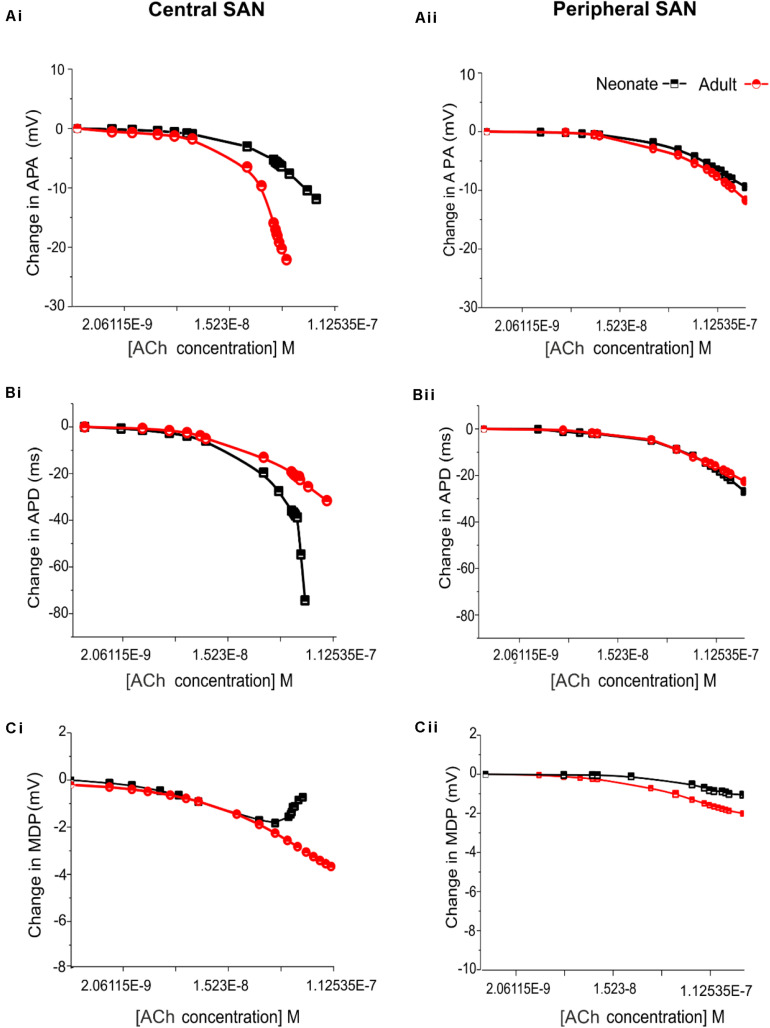
Concentration-dependent effects of ACh on the AP characteristics of rabbit central and peripheral SAN cells for neonate (black squares) and adult (red circles) conditions. **(Ai,Aii)**: Action potential peak amplitude (PA). **(Bi,Bii)**: Action potential duration (APD_50_). **(Ci,Cii)**: Maximal diastolic potential (MDP).

Figure 11 shows the effects of various ACh concentrations on the primary AP characteristics of the neonate cell models (black symbols) compared with the adult cell models (red symbols). Figures 11Ai,Aii shows the changes in AP amplitude; Figures 11Bi,Bii shows the changes in APD_50_; and Figures 11Ci,Cii shows the changes in MDP. For both age groups, all these changes in AP characteristics illustrate that the central cell shows more sensitivity to ACh than the peripheral cell.

### AP Conduction in the Two-Dimensional Tissue Slice Model During SAN Development

**FIGURE 12:**
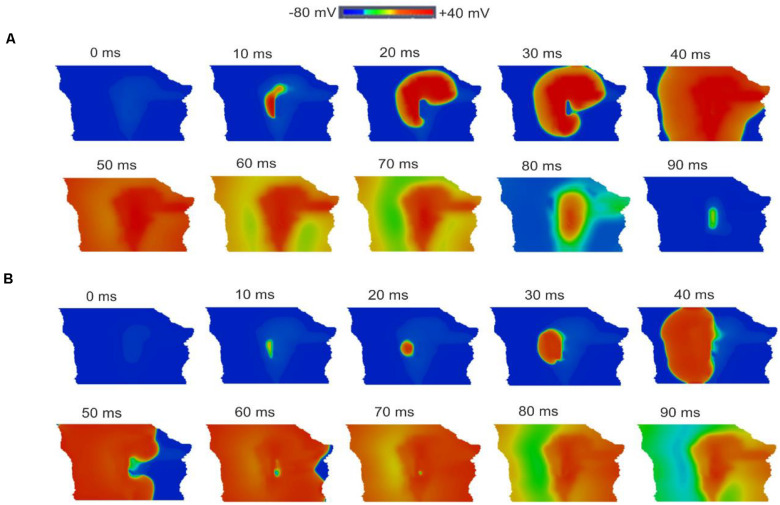
Snapshots of AP initiation and conduction patterns in the 2D model of intact SAN-atrial tissue in the neonate **(A)** and adult **(B)** conditions.

Figures 12A,B shows snapshots of the initiation and conduction patterns of pacemaking APs in the 2D SAN-atrium model for both neonate and adult conditions at different time-points after impulse initiation. In both age groups, the AP propagation displayed a similar conduction sequence, but with a shorter conduction time and thus a greater conduction velocity in the neonate with respect to the adult. In both cases, the AP propagation first started slowly in the center of the SAN. Once initiated, it propagated preferentially from the center toward the periphery of the SAN, and then parallel to the crista terminalis (CT) before entering rapidly into the atrium in a direction toward the atrial septum. The AP conduction was blocked in the block zone, which was encircled by excitation waves from the superior and inferior tissues that surrounded the zone. Such simulated activation and conduction patterns are consistent with experimental observations (Dobrzynski et al., 2005).

**FIGURE 13:**
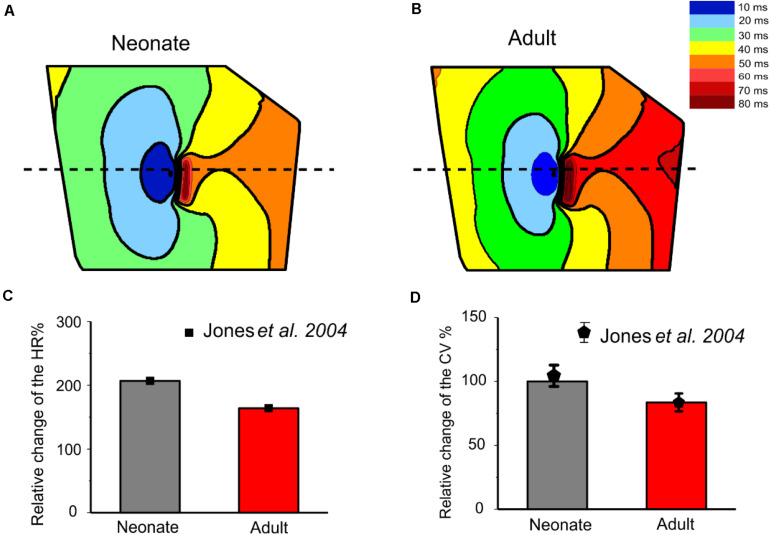
Isochrones of the activation map in 2D rabbit intact SAN-atrial models for both neonatal **(A)** and adult **(B)** conditions. The activation timing in the model was recorded every 10 ms, as illustrated with various colors, which changed from blue at 0 ms to dark red at 80 ms. The bar chart **(C)** shows the reduction of HR and **(D)** the conduction velocity (CV) with developmental maturation in the 2D SAN-atrium, compared with experimental values from Jones et al. (2004).

The isochrones of the activation timing sequence of the SAN-atrium tissue for both conditions are shown in Figures 13A,B. The computed time taken to activate the whole tissue was less in the neonate (55–65 ms) than in the adult (75–95 ms). In association with this, the computed pacemaking CL showed an age-dependent increase, which changed from 290 ms in the neonate to 376 ms in adult tissue, equivalent to a decrease in the heart rate from 207 min^–1^ to 160 min^–1^ in the neonate and adult tissue respectively. This maturation development change in HR was qualitatively close to that observed experimentally from the intact SAN-atrial tissue of guinea pigs (Figure 13C) (Jones et al., 2004; Bai et al., 2017). The measured conduction velocity (CV) was also reduced by developmental maturation in the 2D SAN-atrium model (Figure 13D).

### The Effect of ACh on Two-Dimensional SAN-Atrial Tissue

The single cell simulations showed higher sensitivity of neonatal SAN cells to ACh as compared to the adult, which has also been observed experimentally (Atkins and Marvin, 1989; Yang et al., 2006). Further simulations were conducted to evaluate the functional consequence of ACh on AP conduction in the 2D model of intact SAN-atrium in neonatal and adult conditions.

As at the single cell level, our simulation results implied that when the ACh concentration value was above 8 × 10^–8^ M in neonatal condition, the AP of the central model was suppressed. Therefore, in the 2D model simulations, two physiological values for ACh (3 × 10^–8^ M, 8 × 10^–8^ M) were used.

Supplementary Figure S6 provides snapshots of the AP initiation and conduction sequences in the 2D model for both neonate and adult conditions. With application of ACh ([ACh] = 3 × 10^–8^ M), in both cases, the wave propagation pattern is similar to that seen in the control condition, but with a slower conduction velocity and pacemaking rates (the measured CL changed from 290 ms to 454 ms (by 30%) in the neonate; and changed from 366 ms to 411 ms (by 12.3%) in the adult tissue). The greater effect of the ACh in the neonate as compared with the adult in the tissue simulations was consistent with the simulation results at the single-cell level.

When ACh concentration was increased as shown in Supplementary Figure S7, with [ACh] = 8 × 10^–8^ M, there was a further reduction in the wave propagation velocity within the SAN and atrium tissue, but a more marked increase in the computed pacemaker CL (by 49%). The resulting decreased conduction velocity and increased CL have been observed experimentally for the adult rabbit SAN (Rana et al., 2010). Interestingly, ACh caused a shift of the leading pacemaker site to the peripheral region in the neonatal condition. As the new leading pacemaker was far from the original location, the conduction pathway was altered, leading to differences in the activation timing and conduction velocity in the neonate tissue between the control and ACh conditions (for details see Table 7). The simulated pacemaking shift in response to ACh was consistent with previous experimental observations, in which the leading pacemaker was shifted in response to vagal nerve stimulation (Spear et al., 1979; Shibata et al., 2001; Zhang, 2011).

**TABLE 7:** Simulated CL and averaged CV in control and ACh for both neonate and adult central SAN cell models.

	Cycle length (ms)	Conduction velocity along the SAN (m/s)	HR (b/m)	Reduction of the HR under ACh (%)	HR (b/m) (Jones et al., 2004)
Neonate	290.7	0.30	206.0	0.0	250.0 ± 60.0
Neonate + ACh (3 × 10^–8^ M)	377.21	–	159.1	22.8	–
Neonate + ACh (8 × 10^–8^ M)	569.00	0.18	105.4	49.0	–
Adult	366.21	0.26	163.0	0.0	160.0 ± 20.06
Adult + ACh (3×10^–8^ M)	411.32	–	150.0	8.0	–
Adult + ACh (8×10^–8^ M)	536.1	0.21	111.9	31.13	–

**FIGURE 14:**
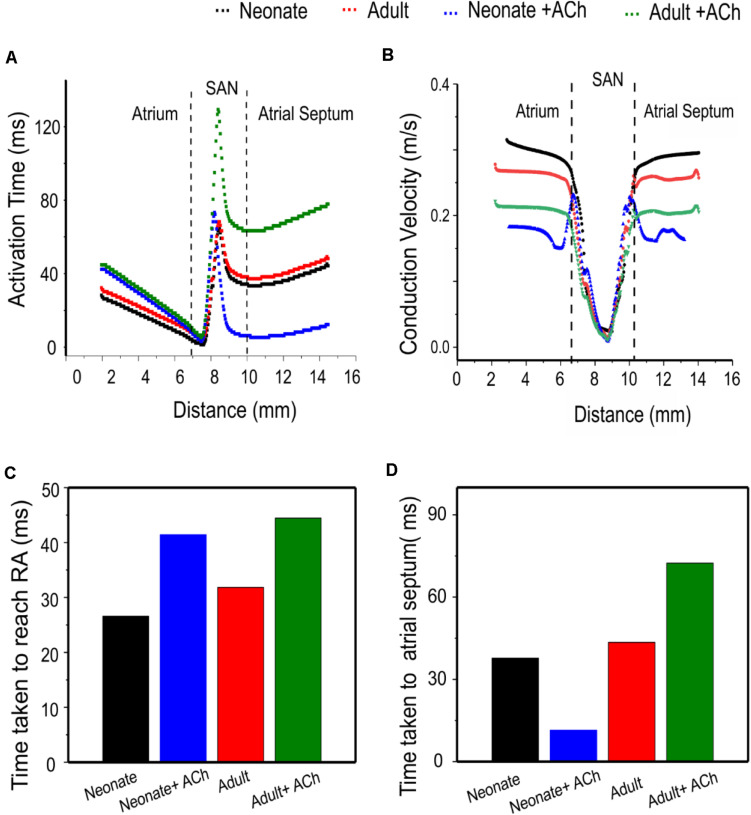
Postnatal development effects on AP conduction across the tissue in the 2D intact SAN-atrium slice. **(A)** Activation time profile and **(B)** conduction velocity of the neonate (black line) as compared with those in the adult (red line) under the effect of WT and ACh at a concentration of [ACh] = 8 × 10^− 8^ M on the neonate (blue line) and adult (green line). **(C,D)** Bar charts showing the increase in time taken for APs to reach the RA, and the relative change of the conduction velocity with maturation development.

Quantitative analyses were also performed to investigate the effect of postnatal development effect on AP activation timing and the AP conduction velocity in the intact SAN-atrial tissue model. Figure 14A plots the measured activation timings from the representative cells across the middle of the 2D neonate tissue for control (black curve) and ACh = 8 × 10^–8^ M conditions (blue line), which were compared with those obtained from the adult tissue in control (red curve) and ACh = 8 × 10^–8^ M conditions (green line). In the adult, the activation timings across the tissue increased. With ACh, the activation time for the neonate was reduced in the direction toward the atrial septum (blue line) due to the shift of leading pacemaking site. The corresponding average conduction velocity across the tissue is shown in Figure 14B. The conduction velocity under control for the neonate condition was found to be greater than that for the adult: approximately 0.30 m/s in the neonate and 0.26 m/s in the adult along the CT. This age-dependent reduction in the CV is in accordance with the increased activation time in the direction toward the CT during developmental maturation. Application of higher ACh concentrations may lead to a further reduced conduction velocity and increased CL and may even impair the SAN to drive the atrial muscle, leading to a “SAN conduction exit block.” Both developmental maturation changes of the activation time and conduction velocity seen in simulations seen in Figures 14C,D were reasonably comparable with the experimental observations from guinea-pig SAN tissue (Jones et al., 2004).

## Discussion

In this study, we have modified the rabbit SAN model developed by Zhang et al. (2000) to simulate the APs of the neonatal SAN based on available experimental data from immature rabbit cells for some major underlying ionic channel currents (details are reported in Tables 1–3). The developed model successfully reproduces the morphology of neonatal SAN APs with their characteristics quantitatively matching experimental data, validating the cell model development (Baruscotti et al., 1996). Using the inclusive method, we showed that the implemented changes in ion channels (as listed in Tables 1–3) due to postnatal development are sufficient to account for the observed changes in pacemaking action potentials between the neonatal and the adult SAN cells. This may have important implications for the diagnosis and treatment of cardiac rhythm disorders in the immature and adult individuals. Using the “exclusive” method, we further analyzed the functional role of individual post-developmental ion-channel remodeling on the APs, by which the major contributor to the fast pacemaking rhythm of the neonate hearts was elucidated. The functional impact of age-related ion-channel remodeling on AP propagation in a 2D model of the intact rabbit SAN-atrium tissue were also investigated. Effects of ACh on pacemaking APs and their conduction were also investigated to elucidate the mechanisms that underlie the post-developmental difference of vagal tone modulation on cardiac pacemaking activities.

The major findings of this study are: (1) at the single cell level, neonatal mathematical models produced higher frequency APs in both the central and peripheral SAN cells with a higher upstroke phase as compared to adult APs. These differences may be attributable to the ion channel remodeling of *I*_*Na*_ and *I*_*f*_ in the developing SAN. The resultant APs at neonatal age showed greater peak amplitude values and smaller APD values, which can be attributed to the post-developmental changes of *I*_*Ca,L*_ and *I*_*NaCa*_; (2) at the tissue level, the post-neonatal changes in cellular APs are reflected by a decrease in the pacemaking rate and AP conduction velocity across the SAN-atrium; (3) acetylcholine had a greater effect on modulating AP pacemaking and conduction in the neonate compared to the adult. A high concentration of ACh in the neonate not only slowed down AP propagation, but also compromised the ability of the SAN to pace and drive the atrium. It could also produce a leading pacemaking site shift to the periphery. These findings together increase understanding of the ion channel mechanisms underlying the different pacemaking activity between the neonatal and the adult SAN cells and their response to acetylcholine, which may be of relevance to understand factors responsible for risk of cardiac arrest (e.g., the “cot death syndrome”) in new-born babies during sleep when the vagal tone is more active.

### Role of Postnatal Ion-Channel Remodeling on Pacemaking Activity

At the cellular level, our simulation results reproduced the development-dependent changes in pacemaking APs and their characteristics, such as the pacemaking rates, between neonatal and adult SAN as observed experimentally (Baruscotti et al., 1996). Further analyses of the role of each individual remodeled ion channel of *I*_*Na*_, *I*_*Ca,L*_, *I*_*f*_, *I*_*Kr*_, *I*_*Ks*_ and *I*_*NaCa*_ by the “exclusive method” elucidated the contribution of each of these channels to the faster pacemaking Aps observed in the neonatal group compared with the adult group.

#### Role of *I*_*Na*_

Our simulation results suggested that (i) the presence of *I*_*Na*_ in the neonate central cells is the one of the major factors responsible for their faster pacemaking activity (i.e., short CL) than that in the adult. This finding is consistent with a previous study (Baruscotti et al., 1996), showing that *I*_*Na*_ was present in rabbit central SAN cells at birth, but fully disappeared by the 40th postnatal day. In the earlier experimental study, the functional role of the current was assessed by applying a Na^+^ channel blocker, TTX, at a concentration of 3 μM and measuring the spontaneous activity of the APs in both the neonatal and adult cells in the SAN. It was found that TTX modified the action potential parameters of the neonatal cells, including a reduction of the upstroke velocity, a decrease in the diastolic depolarization slope from 0.035 to 0.015 V/s, and the consequent slowing down of the pacemaking rate by 60%. However, in the adult cells from the central SAN, *I*_*Na*_ was absent and application of TTX did not show changes in their APs; (ii) the marked developmental maturation change in the *I*_*Na*_ channel properties also contributes to the faster pacemaking APs in the central neonate SAN cells. In the neonatal SAN cells, a noticeable window *I*_*Na*_ current, resulting from the overlap between activation and inactivation curves of the current, provides a depolarizing current in the diastolic phase, accelerating pacemaking APs in neonates. However, with development, the position of the activation curve shifts in the positive direction while the inactivation curve remains, resulting in a reduced overlap of the two curves and hence the window current slowing down the depolarization.

#### Role of *I*_*Ca,L*_ and *I*_*Ca,T*_

Our simulation results showed that the age-related modulation of *I*_*Ca,L*_ has a secondary contribution to the faster pacemaking rate of the neonate. The greater *I*_*Ca,L*_ density in the neonate and the modulation of *I*_*Ca,L*_ kinetics with maturation development (activation curve shifted to the left, steady-state inactivation curve shifted to the right) also play an important role in the high peak value of the APs in the neonate. However, in the adult, although the current density of the *I*_*Ca,L*_ in the central cells was found to be smaller than in the neonate, the opposite shift in activation and inactivation curves during the developmental course resulted in a greater window current in the adult, providing more depolarizing Ca^2+^ current during the depolarization phase of the AP in the adult (Protas et al., 2017), which slowed down the repolarization phase, forming the secondary cause of the fast HR in neonates. Despite the fact that there were recorded changes to the HR between the neonate and adult SAN cells, the absence of developmental changes in *I*_*Ca,T*_ may indicate that this current does not make a major contribution to the different ion currents regulating the APs in central rabbit SAN during development. However, this can vary among different species, as cellular electrophysiology experiments on mice in different age groups showed an age-dependent decline of the heart rate accompanied by a significant decrease of *I*_*Ca,T*_ conductance densities in adult SAN currents (Larson et al., 2013).

#### Role of *I*_*f*_

The present study also demonstrated an association between the age-dependent difference in current density and the modulation of the *I*_*f*_ activation curve in SAN cells, causing a slowing of pacemaking rate during postnatal development. Previous studies demonstrated that the pacemaker current, *I*_*f*_, exhibited an developmental maturation decrease in current density which was assumed to result from a reduction in baseline cAMP levels in the SA node, suggesting that the combined action between the *I*_*f*_ channels and the reduced number of cAMP molecules are the main cause of the shift in the activation curve toward hyperpolarization in the adult group, leaving fewer *I*_*f*_ channels available to initiate diastolic depolarization, a key determinant of the heart rate decrease of the SA node (Accili et al., 1997; Yang et al., 2006).

#### Role of Other Channel Currents

Based on the measurement of gene-expression levels, the great abundance of *I*_*Kr*_, *I*_*Ks*_ and *I*_*NaCa*_ in the neonatal SAN is of relatively small consequence for developmental maturation differences in pacemaking (Vinogradova et al., 2000). In addition, the mechanism of the Na^+^/Ca^2+^ exchange merits study as it is intimately associated with other components of Ca^2+^ homeostasis, such as sarcoplasmic reticular Ca^2+^ stores and release mechanisms, both of which change with postnatal development in other cardiac regions, but not in neonatal SAN (Kaplan et al., 2007). The contribution of the delayed rectifier K^+^ currents and Na^+^/Ca^2+^ exchange current to SAN pacemaking in adults has been previously studied (Tibbits et al., 2002; Janowski et al., 2006; Kaplan et al., 2007), but the role of developmental changes in K^+^ currents in the sinus node remains unexplored (Irisawa et al., 1993; Vinogradova et al., 2000).

### Postnatal Effect on AP Conduction in SAN-Atrial Tissue

At the tissue level, our results show that the combined effect of ion-channel remodeling and the reduced intercellular electrical coupling alters the conduction properties of the APs across the tissue, thereby impairing the function of the SAN with maturation development. The simulation results on the increased conduction time and reduced conduction velocity in the adult tissue are consistent with experimental observations of changed conduction velocity and time taken for the APs to travel toward the RA from guinea pigs (Jones et al., 2004), which demonstrated a link between the progressive increase in the area of SAN tissue lacking the Cx43 protein and the decrease in the intrinsic HR A previous study of intact SAN-atria in guinea pigs during their lifespan (Jones et al., 2004). Without considering the connexin remodeling, ionic-channel remodeling alone resulted in multiple leading pacemaking sites (Supplementary Figure S8), which implied a functional role of connexin remodeling with developmental maturation to ensure a normal pacemaking AP initiation and conduction in the intact SAN-atrium tissue. According to a number of experimental studies (Woods et al., 1976; Yanni et al., 2010; Bai et al., 2017), the electrotonic interaction between the SAN and the atrium affects the pacemaking APs of the SAN cells, leading to a depression in the pacemaking APs. Thus, the magnitude of the CL at the tissue level is greater than that of the isolated single SAN cells.

### Effects of Acetylcholine on the SAN During Developmental Maturation

The chronotropic responsiveness of developing sinoatrial myocytes to acetylcholine (ACh) has been studied (Atkins and Marvin, 1989; Accili et al., 1997). This study elucidated possible mechanisms underlying the increased responsiveness of the neonate SAN cells to ACh. Our simulation results show that the large current density of *I*_*K,ACh*_ in the neonate SAN cells plays a major role in this responsiveness, together with greater densities of *I*_*f*_ and *I*_*Ca,L*_, which play an important secondary contribution to this significant effect of ACh in neonate SAN cells. At both cellular and 2D-tissue levels, a more suppressive effect of ACh on the neonate pacemaking rate in SAN has been observed with increased ACh concentrations, which is comparable with experimental observation (Atkins and Marvin, 1989). Above a critical concentration (>8 × 10^–8^ M), ACh not only slows down the pacemaking rate in SAN cells, but also shifts the leading pacemaking site from the SAN to the periphery (Supplementary Figure S8). In comparison, in the adult tissue, previous studies have demonstrated that the leading pacemaker can be shifted within the SAN in response to vagal nerve stimulation (Spear et al., 1979; Shibata et al., 2001; Zhang, 2011). These studies revealed that the application of specific concentrations of ACh (above 15 × 10^–8^ M) may trigger a leading pacemaker site shift alongside the CT, usually toward the superior vena cava but occasionally toward the interior vena cava. The observed notable increase in the activation timing and decrease in the AP conduction velocity underlies possible conduction failure from the SAN to the atrium, providing a potential mechanistic insight into bradycardia-related dysfunction under conditions of high vagal tone.

### Limitations of the Study

This study was based on mathematical models of the APs of adult rabbit SAN cells, which inherited some limitations that have been described previously in the literature (Zhang et al., 2000; Bai et al., 2017). The model developed for the neonatal SAN cells was based on data from various sources. Specifically, the formulations and the current densities of *I*_*Na*_, *I*_*Ca,L*_ and *I*_*f*_ were based on voltage-clamp data from newborn rabbit SAN cells, while data on *I*_*Kr*_, *I*_*Ks*_, *I*_*K,ACh*_ and *I*_*NaCa*_ were based on data from messenger RNA gene expression or protein levels, as no experimental data on their kinetic or current densities in the SAN cell is available. In model development, we assumed there is a correlation between the gene expression or protein level for certain ion channel subunits and their current densities. However, such correlation may be non-linear and is still unclear. Remodeling of connexin Cx43 was based on data from guinea-pig SAN tissue. Though it is necessary to make clear these limitations, it is an accepted practice in computational modeling to use data from other species when there is a lack of data from the target species (Bai et al., 2017). In addition, our simulations were based on extant data only on ion-channel remodeling, but other factors such as remodeling in Ca^2+^ handling (Allah et al., 2011), ionic homeostasis and energy metabolism, or changes in the phosphorylation levels have not been considered due to lack of available data or because extant mRNA data showed no significant changes (Allah et al., 2011). Such factors may also be of significance in modulation of postnatal pacemaking APs. In addition, the simulation of maturation-related changes in *I*_*Kr*_ and *I*_*Ks*_ was based on the quantitative PCR data from neonatal and adult rabbits (Allah et al., 2011) as there are no direct experimental electrophysiology data that we can use to modify the model. Such data were used to scale conductance, rather than kinetic properties such as the activation and deactivation time constants. Our results (Figure 8) though showed that the postnatal development change in *I*_*Kr*_ and *I*_*Ks*_ hyperpolarized the maximal diastolic potential, but they slowed down, rather than accelerated the pacemaking APs as compared to the adult model. Interpretation of the results on the role of postnatal development related changes in *I*_*Kr*_ and *I*_*Ks*_ in modulating neonatal pacemaking APs needs to be cautious and requires further improvement when more voltage-clamp data become available. However, it is notable that the simulated neonatal pacemaking APs matched well prior experimental data from newborn rabbit SAN cells (Baruscotti et al., 1996; Allah et al., 2011), which validates the model development and justifies the use of parameters for the mathematical model of neonatal rabbit SAN cells.

In simulation of the effects of acetylcholine, it would be valuable also to study the effect of acetylcholine in the presence and absence of adrenergic tone. In the present study, we considered the acetylcholine action only, a computational approach to investigate how ACh alone modulates pacemaking action potentials of isolated SAN cells in neonatal and adult conditions. This is a conventional approach that has been used to investigate the effect of ACh on modulating cardiac pacemaker activity in previous studies by us and others in various conditions, such as sick sinus syndrome and atrial arrhythmogenesis (Butters et al., 2010; Muñoz et al., 2011; Bai et al., 2017). However, this approach presupposes the absence of concomitant adrenergic tone. Further work is warranted to determine the consequences of different levels of sympatho-vagal balance.

The 2D anatomical tissue model considered the anisotropic and electrical heterogeneity of the SAN-atrial tissue, providing valuable insights into the age-related effect on the AP initiation and conduction pathway in the SAN and surrounding tissue. However, further 3D anatomical models that considered tissue anisotropy, heterogeneity and spatial structure might be more useful to investigate the initiation and conduction of APs in control or ACh conditions (e.g., the phenomenon of leading pacemaking site shift). Moreover, the tissue geometry used in the neonatal case here was similar to that in the adult. It has been reported that during the growth of the rabbit, the heart weight and cell diameter increases for the central and peripheral SAN and/or atrial tissue, suggesting that the AP conduction distance may increase with development (Baruscotti et al., 1996; Allah et al., 2011). Further research is therefore required to determine the tissue geometry in neonate rabbit intact SAN-atrial tissue. In addition, in the tissue model we did not consider possible postnatal changes in atrial electrophysiology and used a cell model of the adult rabbit atrial cell due to the lack of experimental data. This limitation demands further improvement when more experimental data become available.

## Conclusion

This simulation study is of significance for three reasons. First, it provides an updated computational model for neonatal rabbit SAN cells. The model has been well validated and can be further used to study the key mechanism responsible for the fast heart rate in neonatal hearts, which may have value for the understanding and treatment of cardiac rhythm disorders in the immature, adult, and aged individuals. Second, the results of the study help understand the dramatic differences in the initiation and conduction of APs at the cellular and tissue levels between neonates and adults. Third, ionic mechanisms responsible for the high sensitivity of neonatal SAN cells to acetylcholine were elucidated. The observation of an amplified effect of ACh in the neonates leading to possible sinus arrest or conduction exit block may be of relevance to cardiac dysfunction in the very young, under situations of high vagal tone.

## Data Availability Statement

The original contributions presented in the study are included in the article/Supplementary Material, further inquiries can be directed to the corresponding author.

## Author Contributions

HZ conceived and designed the study, developed the model, analyzed and interpreted the data, and supervised and wrote the manuscript. AA developed the model, performed the experiments, collected the data, analyzed and interpreted the data, and wrote the manuscript. CT analyzed the data, and edited the manuscript. DW analyzed the data, and edited the manuscript. MB developed the model, analyzed the data, and edited the manuscript. JH developed the model, analyzed and interpreted the data, and wrote the manuscript. All authors contributed to the article and approved the submitted version.

## Conflict of Interest

The authors declare that the research was conducted in the absence of any commercial or financial relationships that could be construed as a potential conflict of interest. The reviewer WG declared a past co-authorship with one of the authors HZ to the handling editor.

## Supplementary Material

Supplementary Figure 1 Sensitivity analyses of the change of the CL as a function of the conductance for *I*_*Na*_ **(A)**, *I*_*Ca,L*_ **(B)**, and *I*_*f*_ **(C)**.

Supplementary Figure 2 The gradient distribution in **(A)** cell capacitance (*C*_*m*_) and **(B)** diffusion (*D*) in both longitudinal and transverse directions of the 2D tissue slice. Figure adapted from Bai et al. (2017). The decline of the Cx43 protein expression in the SAN of rat represented as percentage during developmental maturation **(C)** (Jones et al., 2004).

Supplementary Figure 3 Simulated peripheral SAN action potentials in the neonate (black lines) as compared with the adult (red lines) **(Ai,Aii)** and their underlying time courses of ionic channel currents *I*_*Na*_, *I*_*Ca,L*_, *I*_*f*_, *I*_*NaCa*_, *I*_*Kr*_, and *I*_*Ks*_ **(Bi–Gii)**.

Supplementary Figure 4 Bar chart comparison of the main AP characteristic of peripheral SAN cells: MDP **(A)**, TOP **(B)**, PA **(C)**, d*V*/d*t*_*max*_ **(D)**, reduction of HR **(E)**, and CL **(F)** in the neonate (gray) corresponding to the adult (red).

Supplementary Figure 5 Simulated individual effects of ion-channel remodeling on central SAN action potentials in the adult and the underlying ionic currents **(Ai,Aii)** *I*_*Na*_, **(Bi,Bii)** *I*_*Ca,L*_, **(Ci,Cii)** *I*_*f*_, **(Di,Dii)** *I*_*Kr*_, **(Ei,Eii)** *I*_*Ks*_ and **(Fi,Fii)** *I*_*NaCa*_.

Supplementary Figure 6 ACh effect at a concentration of 3 × 10^–8^ M on the propagation of the SAN and surrounding area of the neonate **(A)** and adult **(B)** rabbit 2D tissue. Snapshots of the activation pattern at various timings are shown.

Supplementary Figure 7 ACh effect at a concentration of 8 × 10^–8^ M on the propagation of the SAN and surrounding area of the neonate **(A)** and adult **(B)** rabbit 2D tissue. Snapshots of the activation pattern at various times are shown.

Supplementary Figure 8 Multi-leading pacemaking sites resulting from ion-channel remodeling in the absence of connexin remodeling. Snapshots of the activation pattern at various timings are shown.

Supplementary Table 1 AP characteristics of the neonate and adult peripheral SA node cell models.
