# Supplementary material for: Mechanistic Insights Into the Reduced Pacemaking Rate of the Rabbit Sinoatrial Node During Postnatal Development: A Simulation Study
Source: Front Physiol. 2020 Nov 20;11:547577. doi: 10.3389/fphys.2020.547577 (PMC7715043; doi:10.3389/fphys.2020.547577)
Supplement: Supplementary Figure 1 — Sensitivity analyses of the change of the CL as a function of the conductance for INa (A), ICa,L (B), and If (C). [file Data_Sheet_1.docx]

**Supplemental Figures and Table**


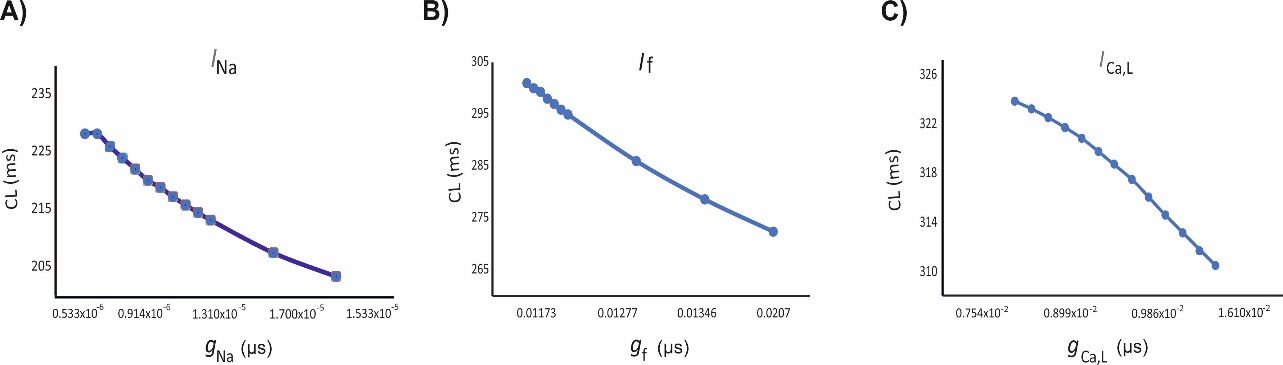


**Figure S1.** Sensitivity analyses of the change of the CL as a function of the conductance for *I*_Na_,(A) *I*_Ca,L_ (B) and *I*_f_ (C) .


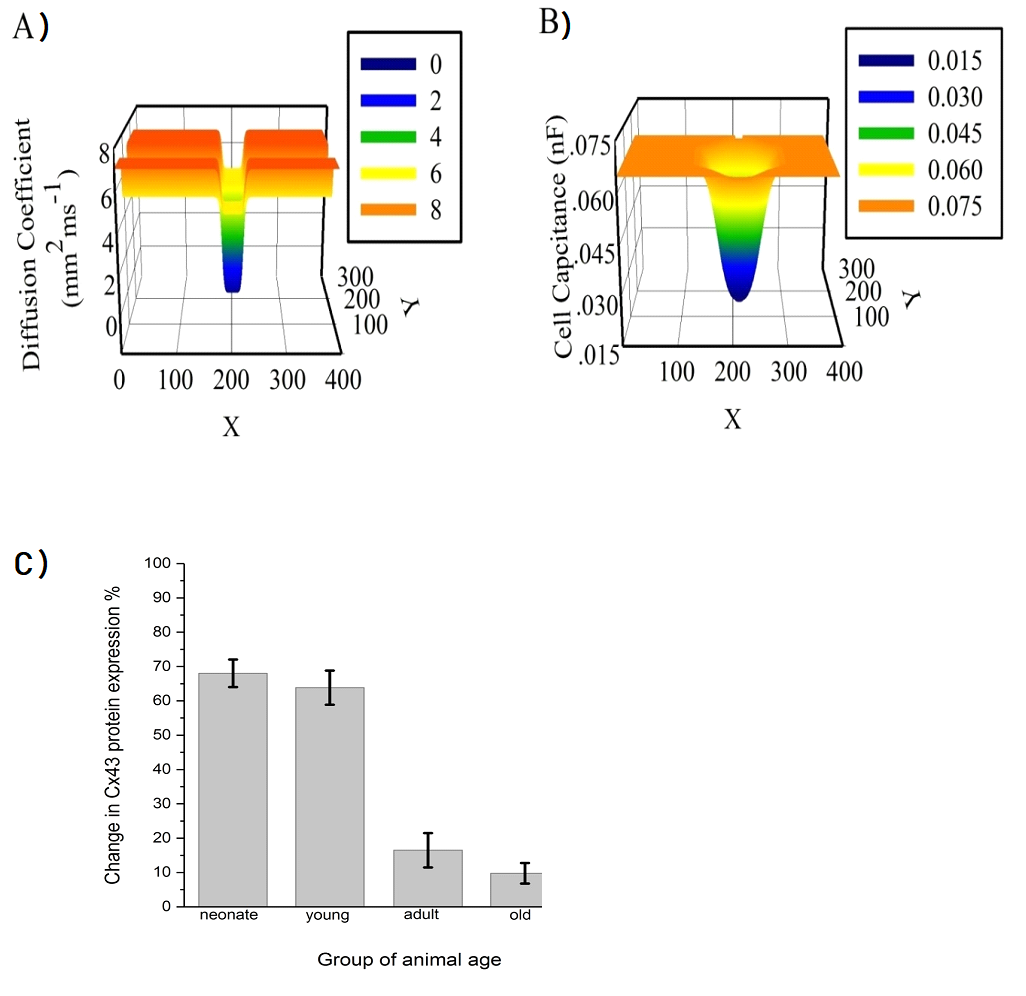
**Figure S2.** The gradient distribution in (A) cell capacitance (*C*_m_) and (B) diffusion (*D*) in both longitudinal and transverse directions of the 2D tissue slice. Figure adapted from [28].The decline of the Cx43 protein expression in the SAN of rat represented as percentage during developmental maturation.(C) [22]


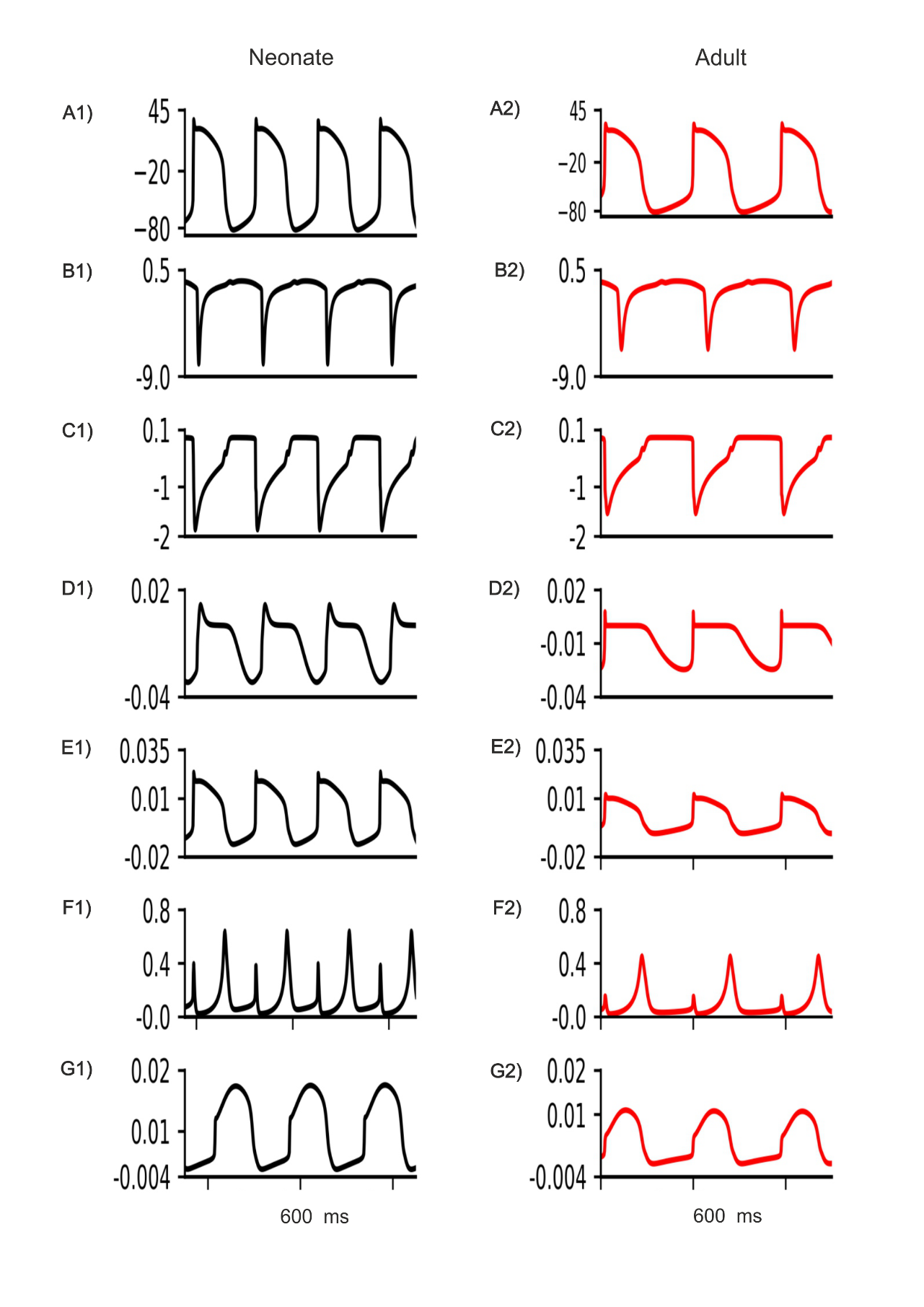


**Figure S3**. Simulated peripheral SAN action potentials in the neonate (black lines) as compared with the adult (red lines) (Ai-Aii) and their underlying time courses of ionic channel currents *I*_Na_, *I*_Ca,L_, *I*_f_, *I*_NaCa_, *I*_Kr_ and *I*_Ks_ (Bi-Gii).

^
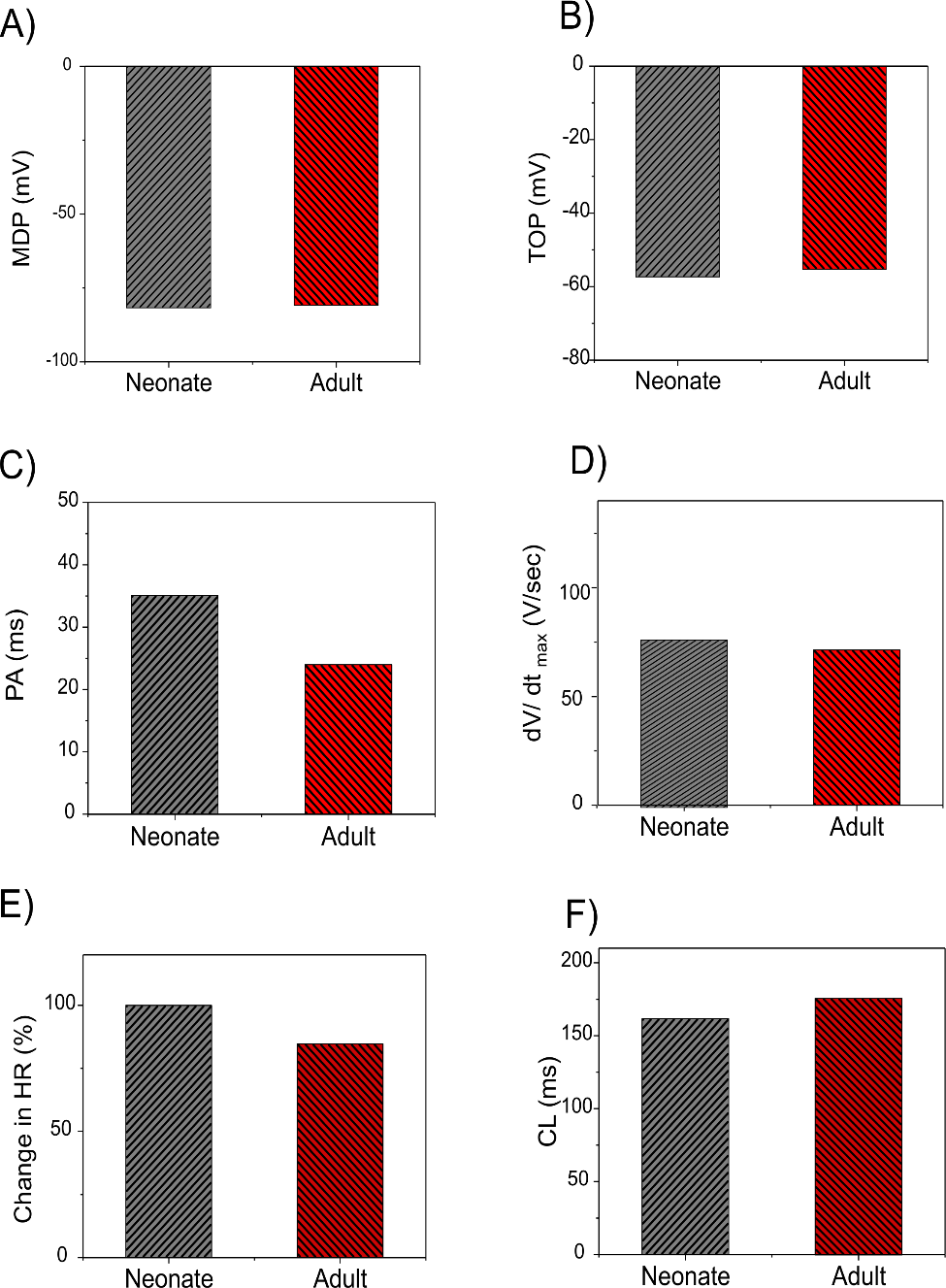
^

**Figure S4**. Bar chart comparison of the main AP characteristic of peripheral SAN cells: MDP (A), TOP (B), PA (C), d*V*/d*t*_max_ (D), reduction of HR (E), and CL (F) in the neonate (grey) corresponding to the adult (red).

**Table S1**. AP characteristics of the neonate and adult peripheral SA node cell models.

|  | References | PA (mV) | CL (ms) | MDP (mV) | dV/dt_max_ (V/s) |
| --- | --- | --- | --- | --- | --- |
| Neonate | Our model | 31.69 | 156.23 | -78.45 | 72.83 |
| Adult | Zhang *et al*.[26] | 23.07 | 175.39 | -77.96 | 70.16 |


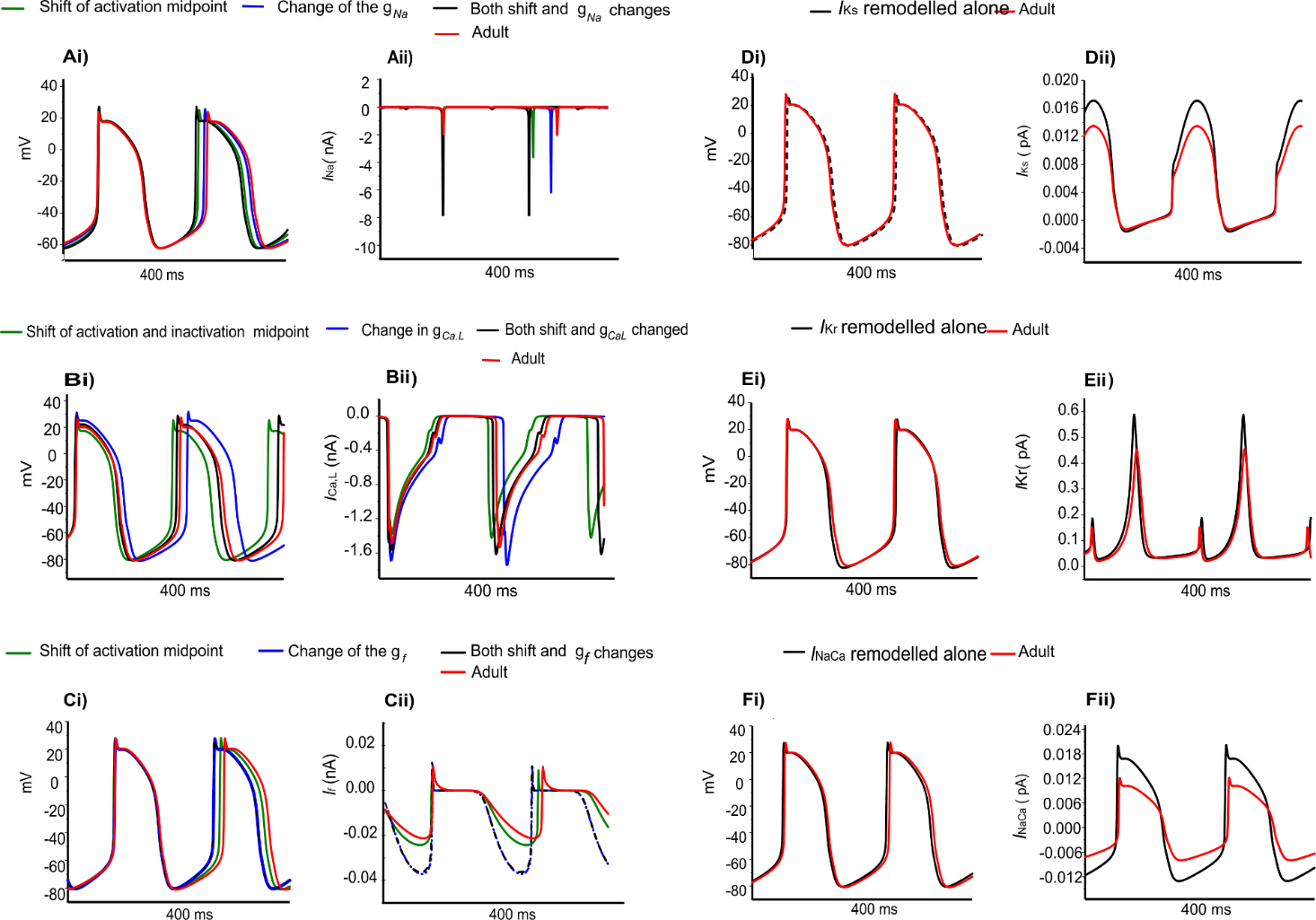


**Figure S5.** Simulated individual effects of ion-channel remodelling on central SAN action potentials in the adult and the underlying ionic currents (Ai-Aii) *I*_Na_, (Bi-Bii) *I*_Ca,L_, (Ci-Cii) *I*_f_, (Di-Dii) *I*_Kr_, (Ei-Eii) *I*_Ks_ and (Fi-Fii) *I*_NaCa_


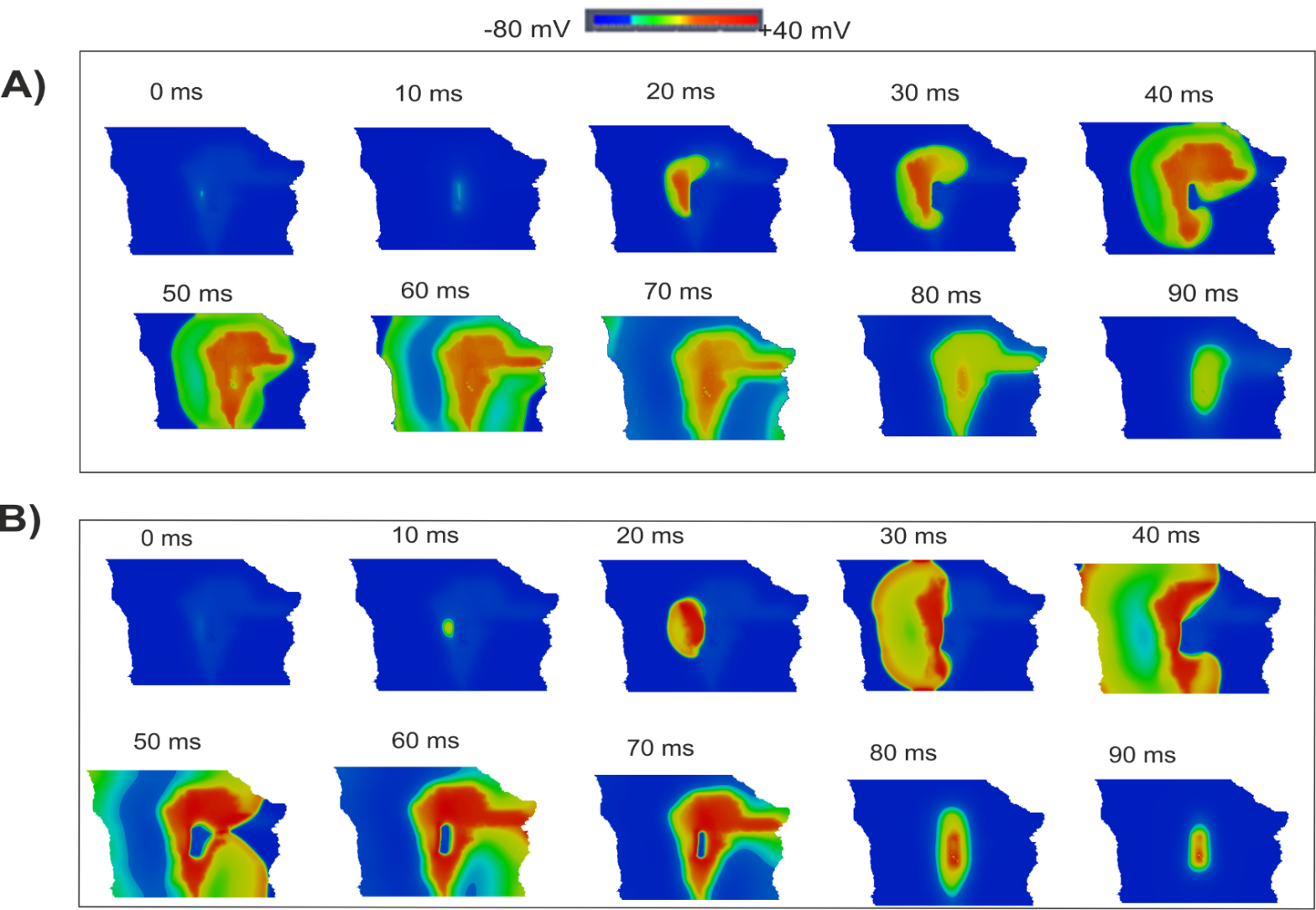


**Figure S6.** ACh effect at a concentration of 3×10^-8^M on the propagation of the SAN and surrounding area of the neonate (A) and adult (B) rabbit 2D tissue. Snapshots of the activation pattern at various timings are shown.


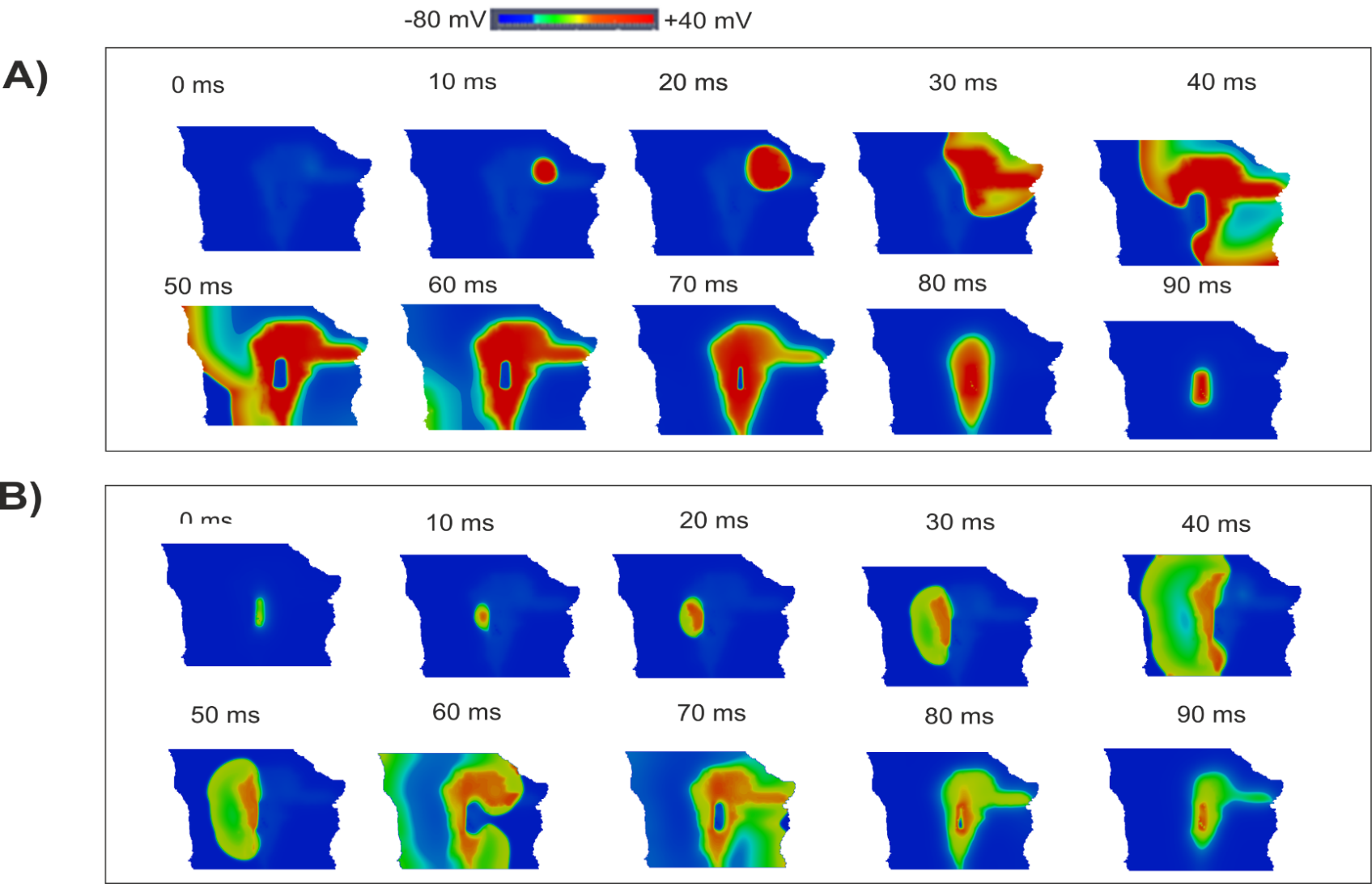


**Figure S7.** ACh effect at a concentration of 8×10^-8^M on the propagation of the SAN and surrounding area of the neonate (A) and adult (B) rabbit 2D tissue. Snapshots of the activation pattern at various times are shown.


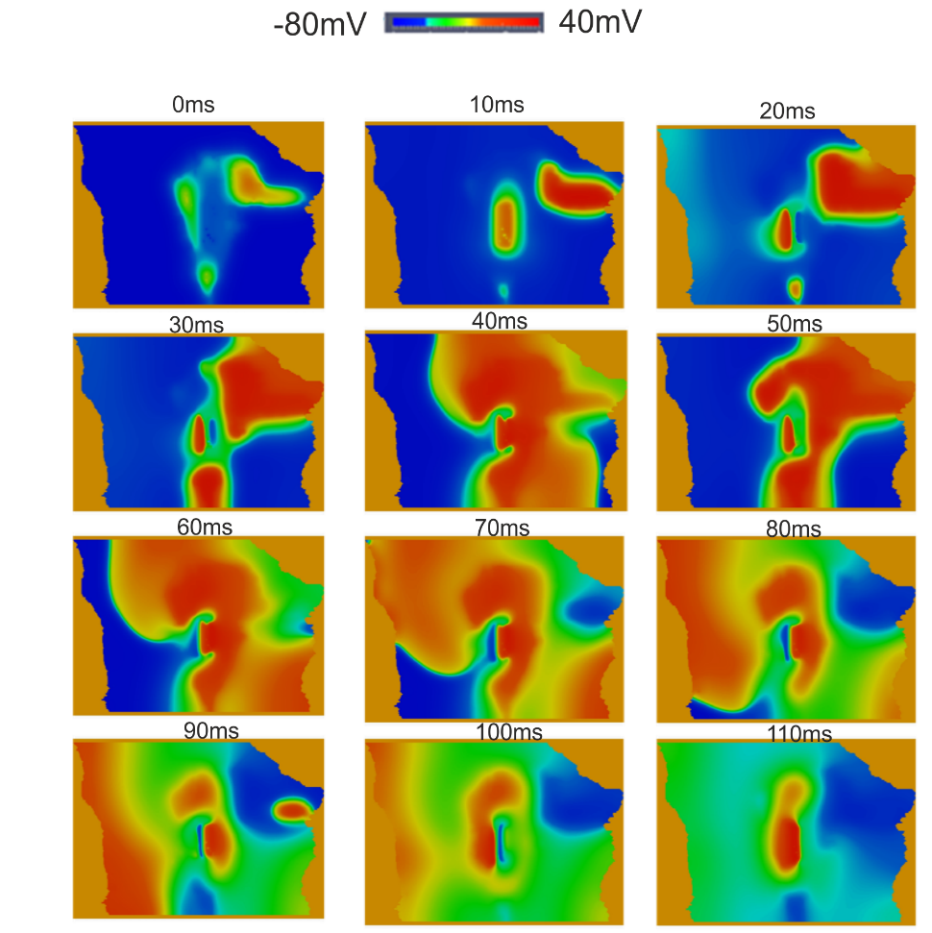


**Figure S8.** Multi-leading pacemaking sites resulting from ion-channel remodelling in the absence of connexin remodelling. Snapshots of the activation pattern at various timings are shown.

## References

22. Jones SA, Lancaster MK, Boyett MR. Ageing-related changes of connexins and conduction within the sinoatrial node. J Physiol [Internet]. 2004 Oct [cited 2017 Jul 26];560(2):429–37. Available from: http://doi.wiley.com/10.1113/jphysiol.2004.072108

26. Zhang H, Holden A V, Kodama I, Honjo H, Lei M, Varghese T, et al. Mathematical models of action potentials in the periphery and center of the rabbit sinoatrial node. Am J Physiol Hear Circ Physiol. 2000;279(2000):H397–H421.

28. Bai X, Wang K, Yuan Y, Li Q, Dobrzynski H, Boyett MR. Mechanism underlying impaired cardiac pacemaking rhythm during ischemia. A simulation study. the American Institute of Physics. 2017 Chaos **27**, 093934. doi: 10.1063/1.5002664
